# Unified platform for multiplex immunofluorescence across liver tissues and engineered models

**DOI:** 10.1136/egastro-2026-100379

**Published:** 2026-04-30

**Authors:** Bianca Franco Leonardi, Guo Yin, Natalja Amiridze, Tian Lan, Yeni Ait Ahmed, Hilmar Berger, Frank Tacke, Marlene S Kohlhepp, Adrien Guillot

**Affiliations:** 1 Department of Hepatology and Gastroenterology, Campus Virchow-Klinikum and Campus Charité Mitte, Charité - Universitätsmedizin Berlin, Berlin, Germany; 2 Department of Physiology and Biophysics, Institute of Biomedical Sciences, University of Sao Paulo, São Paulo, Brazil; 3 Department of Gastroenterology, West China Hospital, Sichuan University, Chengdu, Sichuan, China

**Keywords:** Liver Diseases, Organoids, Microphysiological Systems, Microscopy, Fluorescence, Image Processing, Computer-Assisted

## Abstract

**Background:**

Multiplex immunostaining combined with digital image analysis has become a central tool in hepatology research because it allows for the simultaneous visualisation and spatial mapping of multiple cellular markers within a single tissue section, providing critical insights into the complex, heterogeneous microenvironments of liver diseases like cholangiopathies, hepatocellular carcinoma, metabolic dysfunction-associated steatohepatitis or hepatic fibrosis. Nevertheless, significant barriers prevent the widespread adoption of most multiplexing platforms, including the technological complexity that necessitates specialised equipment and personnel. Additionally, several technologies remain incompatible with standard laboratory workflows and archival formalin-fixed paraffin-embedded (FFPE) tissue or in vitro culture samples.

**Methods:**

We developed a customised immunofluorescence workflow based on sequential cycles of antibody stripping to generate multiplexed digital images from FFPE liver sections, intrahepatic cholangiocyte organoids and primary mouse liver cells cultured either in conventional two-dimensional systems or within a biliary niche-on-a-chip platform. Subsequent image processing and channel alignment were performed using an in-house and open-source, Python-based software tool.

**Results:**

We demonstrate the functionality of a multiplex immunofluorescence protocol that can be readily adapted to a broad range of archival tissues and primary cell-based samples. This workflow allows the visualisation of antigen-expressing cells and protein–protein interactions using proximity ligation assays. This study also details the technical considerations necessary for its rapid integration into routine workflows in virtually any laboratory equipped for conventional immunohistochemistry.

**Conclusions:**

This methodology integrates multiplex immunofluorescence into standard laboratory workflows, thereby enabling researchers to overcome challenges such as technological complexity and cost for transitioning from conventional to multiplexed microscopy.

**Figure:**
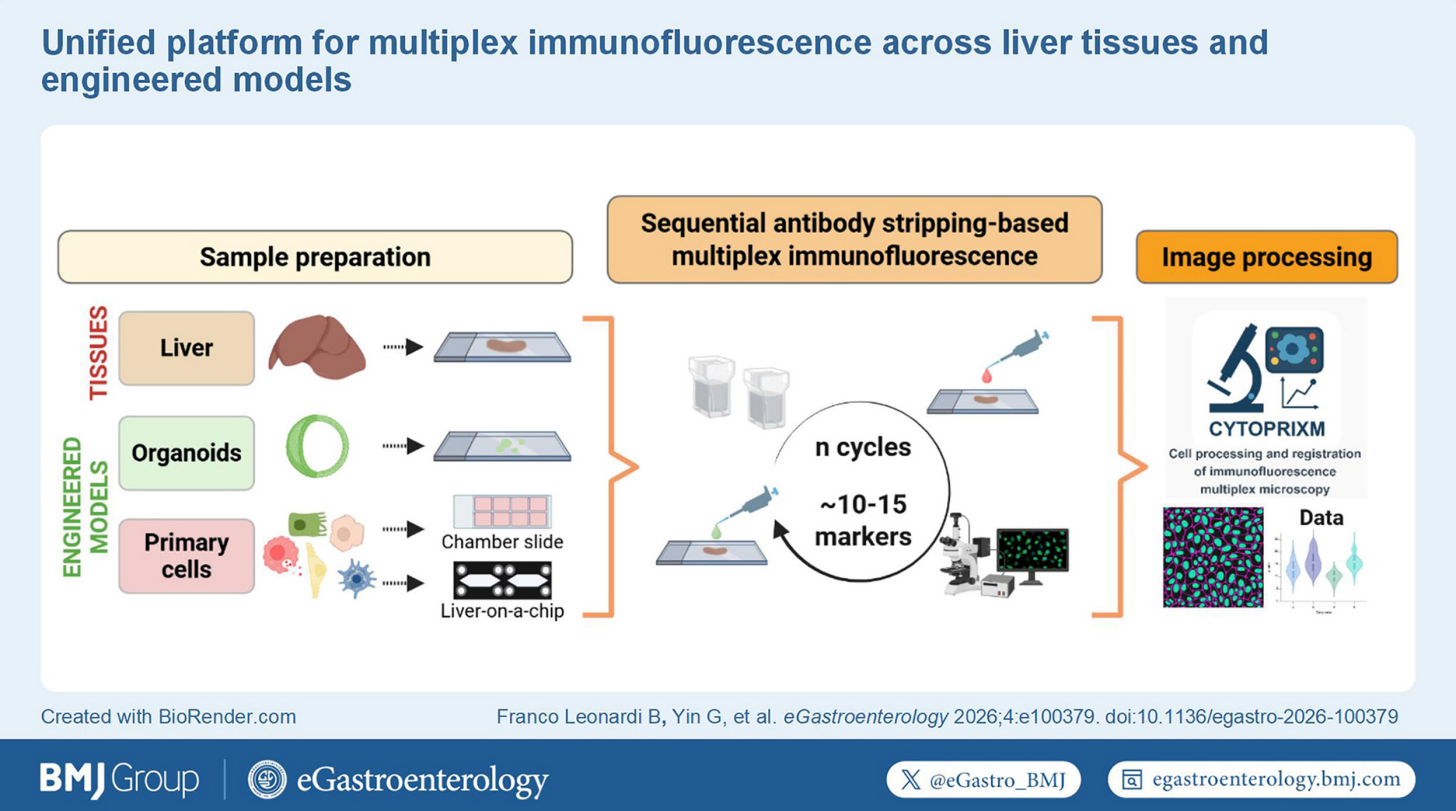
Visual abstract

**WHAT IS ALREADY KNOWN ON THIS TOPIC**

- A wide range of methods now enable multiplex immunostaining using fluorochrome-conjugated, metal-conjugated or probe-conjugated primary or secondary antibodies. However, their broader adoption is often hindered by substantial challenges, including operational and analytical complexity that requires specialised personnel, expensive equipment, high reagent costs and limited flexibility in panel design.

**WHAT THIS STUDY ADDS**

- We present a detailed description of our technical approaches and quality controls for implementing multiplex immunofluorescence across a range of multicellular contexts, including tissue specimens and primary cell-derived, engineered in vitro systems. We also introduce CytoPrixm, an in-house, open-source software tool that standardises the preprocessing of multidimensional images prior to downstream analyses.

**HOW THIS STUDY MIGHT AFFECT RESEARCH, PRACTICE OR POLICY**

- In the era of single-cell omics, the standardisation of multiplex immunofluorescence as described here offers an accessible strategy for large-scale phenotyping while enabling high-quality, image-based mechanistic analyses with minimal changes in standard operating procedures and marginal reagent costs. Within liver disease research, this approach facilitates the investigation of disease-driving processes and cellular crosstalk networks, thereby accelerating progress in the field.

## Introduction

 Liver diseases are among the leading causes of global mortality, and their incidence continues to rise worldwide.^1^ A wide range of conditions with different aetiologies can affect the liver, including acute diseases such as acute viral hepatitis and drug-induced liver injury, as well as chronic disorders such as chronic viral hepatitis, metabolic dysfunction-associated steatotic liver disease (MASLD), metabolic dysfunction-associated steatohepatitis (MASH) and alcohol-associated liver disease. In addition, cholestatic diseases that impair the bile ducts and bile flow, such as primary biliary cholangitis (PBC) and primary sclerosing cholangitis (PSC), are also of major relevance.^2^ Despite significant advances in understanding the pathogenesis of various liver diseases, therapeutic options remain limited in several cases. Moreover, many of the available pharmacological treatments show insufficient efficacy due to the pronounced heterogeneity of disease phenotypes and patient-specific response to therapies.^3 4^ This situation is most certainly calling for in-depth characterisation of single cell resolved pathomechanisms at the level of individual patients. In line with recent technological advances, clinical and research practices, including in hepatology, are rapidly changing.^5^

The liver is a central metabolic organ primarily composed of hepatocytes, along with other essential cell types such as Kupffer cells (KCs), liver endothelial cells (ECs), hepatic stellate cells (HSCs) and cholangiocytes in addition to patrolling immune cells from the circulation.^6^ These diverse cell populations form a dynamic network of complex interactions that not only maintain organ homeostasis but also actively contribute to the initiation, progression and resolution of liver diseases.^7^,^9^ Additionally, liver functions are distributed across tightly regulated metabolic and immunological niches, generating a spatially organised microenvironment. Consequently, advances in understanding the functional and spatial liver landscape hold significant clinical relevance.^10 11^

Single-cell and spatial multiomics approaches represent a rapidly expanding field that is transforming our understanding of cellular biology in health and disease.^12^ However, standalone techniques fail to capture the full complexity of liver pathogenesis. A more profound and reliable understanding of disease progression can be achieved through the integration of methods that resolve cellular heterogeneity and novel molecular states, while ensuring in vivo spatial and morphological validation.^13 14^ A widely used approach for exploring proteins in their native tissue context is multiplex immunohistochemistry or immunofluorescence, which allows simultaneous labelling of multiple proteins within the same tissue section. Furthermore, due to advances in the field, the techniques, formerly limited to the detection of only a few antigens, have scaled to higher-plex levels, allowing the labelling of more than 60 targets within the same tissue.^15 16^ These methods include iterative staining and image acquisition steps followed by chemical or enzymatic removal of antibodies or their signals (eg, cyclic immunofluorescence, CycIF); laser- or ion beam-based approaches coupled to mass spectrometers for the analysis of metal-labelled antibodies (cytometry by time-of-flight); and oligonucleotide barcode-based technologies, such as Co-Detection by Indexing (CODEX/PhenoCycler).^17^

Multiple parameters must be considered when selecting a method, including the nature of the sample (eg, formalin-fixed paraffin-embedded (FFPE) vs frozen), the number of markers required, processing time, technical sensitivity and costs.^18^ Indeed, most multiplexed techniques remain demanding in terms of personnel specialisation and require significant human and material resource investments. For example, when a limited number of markers (10–20 antibodies) is needed for hypothesis validation in a substantial cohort with large tissue areas of interest, it may be deemed excessive to invest in complex and costly methodologies. In this study, we elaborate on an updated workflow for antibody stripping-based multiplex immunofluorescence (mIF) protocol for engineered (in vitro) and native (in situ) samples. Additionally, we provide new software solutions for the preprocessing of multidimensional imaging data, designed to enhance the time efficiency of the subsequent analytical workflows.

## Materials and methods

### Liver FFPE sample preparation

Livers from healthy C57BL/6J mice and from models of acute liver injury induced by a single intraperitoneal carbon tetrachloride (CCl_4_) injection (0.5 mL CCl_4_/kg body weight in corn oil) 24 hours prior to sacrifice, and progressive cholangiopathy in multidrug resistance 2 (*Mdr2*) knockout mice (24-week-old) were retrieved. After collection, tissues were immediately fixed in 4% paraformaldehyde (PFA, pH 7.4, Morphisto, Frankfurt, Germany) for 48 hours, dehydrated and embedded in paraffin. Sections of 2 µm thickness were prepared for immunostaining. It is strongly recommended to store paraffin blocks and sections at 4°C to preserve tissue quality, and to ideally perform the mIF within a relatively short time (<6 months) after cutting sections. Slides were deparaffinised in xylene, then rehydrated through graded ethanol solutions (95%, 80%, 70% and 50%; 2 min each) and finally transferred to distilled water. Human liver sections were prepared similarly.

### Proximity ligation assay

The CD8/major histocompatibility complex (MHC)-I proximity-ligation-assay was performed on 2 µm-thick FFPE sections of human liver according to the protocol provided by the manufacturer (PPI.TCR01.FR.100, Navinci, Uppsala, Sweden), with slight modifications, that is, for antigen retrieval using a water bath set at 98°C for 25 min. An EDTA solution at pH 9.0 (Monosan HIER Tris-EDTA Buffer pH 9.0, MON-APP164; Monosan, Uden, The Netherlands) was used at this step, and for subsequent antigen retrieval steps, the Universal HIER Antigen Retrieval Reagent 10X (Abcam, Cambridge, UK) was used. After image acquisition, the sections were stripped as described previously,^19^,^21^ prior to subsequent immunofluorescence and stripping cycles.

### Intrahepatic cholangiocyte fixation

Mouse intrahepatic cholangiocyte organoids (mICOs) were incubated in Cultrex organoid harvesting solution (Bio-Techne GmbH, Wiesbaden, Germany) for 30 min at 4°C to remove Matrigel, followed by two washes with cold phosphate-buffered saline (PBS). Organoids were then fixed in 4% PFA for 1 hour at room temperature (RT) and washed twice with cold PBS. Next, organoids were dehydrated and stained with 0.5% eosin dissolved in 96% ethanol, embedded in paraffin and sectioned for immunostaining. The organoids were not centrifuged to prevent alterations but instead left to settle under gravity. Alternatively, mICOs were dissociated using Cultrex organoid harvesting solution to generate single cell suspensions of biliary cells, prior to seeding onto collagen-coated chamber slides or biochips.

### Fixation of adherent cells in the chamber and liver-on-a-chip culture systems

After 1–3 days of culture, the culture medium was gently removed, and the samples were immediately fixed by adding ice-cold methanol and incubating at −20°C for 7 min. The methanol was then removed, and the cells were post-fixed with 4% PFA in PBS for 10 min at RT. Following fixation, the samples were washed three times with PBS to remove residual fixative.

### Antigen retrieval and signal enhancer

After deparaffinisation, tissue sections underwent antigen retrieval by immersing the slides in a prewarmed antigen retrieval solution, either citrate (Monosan HIER Citrate Buffer pH 6.0 (10X)) or Universal HIER Antigen Retrieval Reagent 10X (Abcam), for mouse or human liver sections, respectively, diluted to 1× in deionised water. This step was performed in a water bath set at 98°C for 20 min, followed by a cooling period, in this same buffer, at RT on the bench for at least 30 min and washing three times with PBS for 2 min each. Subsequently, a water-repellent barrier was drawn, and one to two drops of Image-iT FX Signal Enhancer (Cell Signaling Technology, Danvers, Massachusetts, USA), reagent were applied to each section. After incubation for 30 min at RT, the slides were washed three times with PBS for 2 min each before proceeding with subsequent staining procedures.

### Non-specific antigen blocking

The final step before incubation with the primary antibody was the blocking of non-specific antigens, which was performed uniformly across all sample types by incubating the samples with 2% normal goat serum (Thermo Fisher, Waltham, Massachusetts, USA) for 1 hour at RT. Following this, the samples were washed three times with PBS for 2 min.

### Antibody incubation

All samples, except organoid FFPE samples, were incubated overnight at 4°C with primary antibodies diluted in PBS containing 1% bovine serum albumin (BSA). For organoid FFPE samples, primary antibodies were diluted in PBS containing 0.1% BSA. The following day, excess antibodies were removed by washing once with PBS-T (PBS containing 0.1% Tween 20) for 2 min, followed by two additional washes with PBS. Samples were then incubated for 1 hour at RT with secondary antibodies selected according to the host species of the primary antibodies used in each cycle. The secondary antibody mixture was prepared in PBS containing 1% BSA; for organoid FFPE samples, PBS containing 0.1% BSA was used instead. After incubation, samples were washed again (first with PBS-T then two times with PBS). A list of primary and secondary antibodies used in this study is provided in online supplemental tables 1 and 2, respectively.

### Nucleus staining

Following the secondary antibody step, the samples were incubated with a 2 µg/mL solution of 4′,6-diamidino-2-phenylindole (DAPI; dissolved in PBS) for 5 min at RT to stain cell nuclei and subsequently washed three times with repeated changes of distilled water for 2 min each. The nuclei of living cells were stained by a 5 min incubation using Hoechst 33342 (Thermo Fisher), followed by washing with PBS.

### Antibody elution

At the end of each antibody staining and imaging cycle, antibodies were eluted to allow the initiation of a new cycle using the previously described 2-mercaptoethanol/sodium dodecyl sulphate (SDS) method.^19 22^ Briefly, samples were incubated at 56°C for 1 hour in the antibody stripping buffer containing 62.5 mM Tris-HCl (pH 6.8), 2% (w/v) SDS and 114.4 mM β-mercaptoethanol prepared in distilled water. Following stripping, samples were washed three times with PBS-T for 20 min each under gentle orbital agitation. Another step of antigen retrieval was then performed prior to the next round of primary antibody incubation.

### Tissue and conventional two-dimensional primary cell culture image processing using CytoPrixm

Images shown in this study were generated as follows and as previously described.^20^ After image acquisition, czi files containing tile scans were subjected to a shading correction with a shading reference image. Then, images were stitched, and a rolling-ball background subtraction algorithm was applied.^23^ Exported TIFF files of each marker acquired on the same day were then combined into a stack, and consecutive stacks from a single sample were concatenated into a hyperstack, then registered using DAPI as a reference. For all tissue-derived and conventional two-dimensional cell culture-derived images, those postacquisition processing steps were carried out using CytoPrixm, an in-house, open-source software tool that we provide to enable rapid preparation of multidimensional images for downstream analyses as described in this study (online supplemental material). CytoPrixm can be downloaded at https://doi.org/10.5281/zenodo.19059527 and user's instructions are provided as online supplemental material.

### Image preparation for the liver-on-a-chip

Due to inherent physical properties of the membrane on which the cells adhere, tile scan images were acquired as a z-stack. Prior to all steps described above, orthogonal projections with weighted average were performed in ZEN (Zeiss, Jena, Germany). Images were then exported without compression.

Additional technical details are provided in the online supplemental methods.

## Results

### Sequential multiplex immunofluorescence generic workflow for formalin-fixed, paraffin-embedded tissue samples

**Figure 1:**
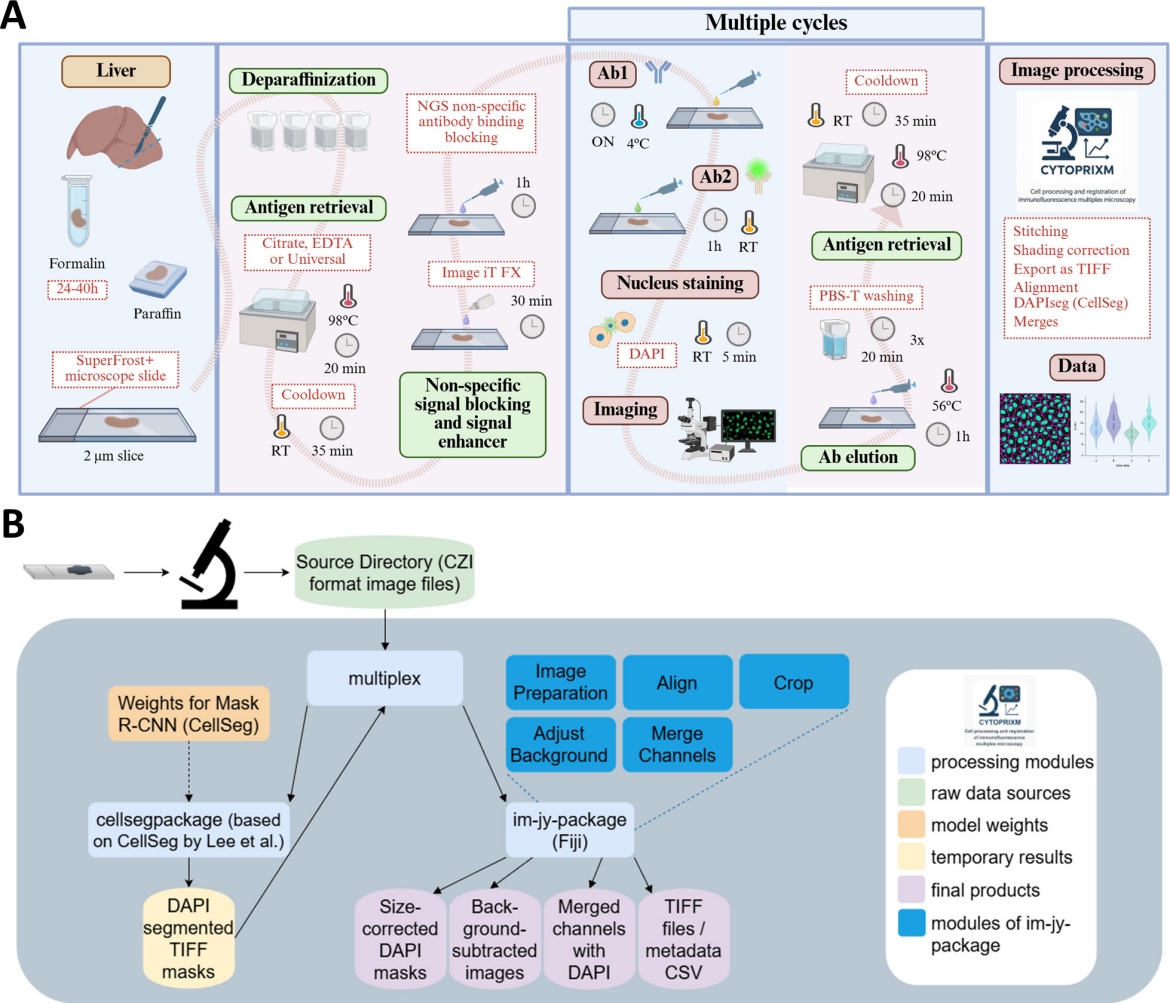
Sequential multiplex immunofluorescence and CytoPrixm software overviews. (**A**) Generic workflow of multiplex immunostaining applied to the phenotypic characterisation of cells in formalin-fixed, paraffin-embedded tissues, illustrating the sequential steps from sample preparation to the acquisition of final analytical results. (**B**) CytoPrixm software component diagram. The pipeline is organised into three functional blocks: processing modules (bright blue), inputs (green) and outputs (yellow and purple). Solid arrows indicate data flow, while dashed arrows represent configuration or weight dependencies. Created with BioRender.com. Ab, antibody; DAPI, 4′,6-diamidino-2-phenylindole; NGS, normal goat serum; ON, overnight; PBS-T, phosphate-buffered saline containing 0.1% Tween 20; R-CNN, region-based convolutional neural network; RT, room temperature.

Figure 1A presents the workflow of the method, encompassing sample preparation, sequential immunofluorescence and image preparation from FFPE tissue sections. Briefly, FFPE liver samples are sectioned using a microtome at a thickness of 2 µm, and placed onto adhesive microscopy slides and dried. Subsequently, the slides undergo deparaffinisation in xylene, followed by washes in decreasing concentrations of ethanol and in distilled water. Next, the antigen retrieval step is performed by incubating the samples in the preheated buffer of choice such as citrate (pH 6.0), EDTA (pH 9.0) or Universal Antigen Retrieval buffer. The choice of the antigen retrieval solution must be tested prior to performing mIF and is antibody dependent. Next, Image-iT FX signal enhancer is applied to improve signal-to-background ratio. Non-specific antibody binding is blocked with normal goat serum. The samples are then ready for the staining cycles. First, the slides are incubated with the first mixture of primary antibodies, usually overnight at 4°C. On the following day, the slides are washed and then incubated with the secondary, fluorophore-conjugated antibodies. Despite adding an extra step in the protocol, the use of secondary antibodies is preferred as this provides signal amplification and panel flexibility compared with preconjugated primary antibodies. After nuclear staining, the samples are mounted in an aqueous mounting medium for 1–2 min, and a representative area of the tissue is selected for image acquisition. Exposure times can be adapted as appropriate per antibody and per day but should remain constant among the same batch of samples. After imaging is completed, the slides are immediately immersed in distilled water at RT (usually 5–10 min until the coverslip slides down when the microscopy glass is gently pulled out of the water solution). Next, the slides are incubated in the antibody stripping solution, then thoroughly washed under orbital agitation. The efficiency of the stripping may be verified by using the same imaging parameters as prior to antibody stripping. The next staining cycle is then initiated, starting from the antigen retrieval step, and this process is repeated until all markers of interest have been stained. The antibody sequence can be modified from one day to another based on preliminary observations. Tissues in good condition can usually stand 8–12 cycles without any issue when the experimenter is sufficiently trained.

### An open-source platform designed to accelerate multidimensional image preparation for analysis

Due to the number of cycles and a potentially large panel of markers that can be incorporated into each batch of samples, sequential imaging generates a substantial volume of imaging data, which requires careful processing before it can be subjected to formal analyses. This may demand a considerable investment of time and dedication from the researcher. To overcome this limitation, we developed a new software tool, CytoPrixm, which enables faster preparation of multiplex-derived images for subsequent analytical steps. In addition, the use of this platform helps reduce potential human errors associated with the organisation and initial processing of the images. In figure 1B, we introduce the overall algorithm and packages contained within our software CytoPrixm. This software combines several open source and freely available codes and algorithms (online supplemental tables 3–6, online supplemental methods and online supplemental materials), which we have streamlined and optimised so that non-experts can prepare mIF-derived images in a timely manner with minimal training. The workflow comprises a graphical user interface that integrates tightly with state-of-the-art imaging software (Fiji/ImageJ) and a previously published deep-learning based nucleus segmentation software (CellSeg).^24^

### Investigation of the liver cellular landscape in FFPE samples

**Figure 2:**
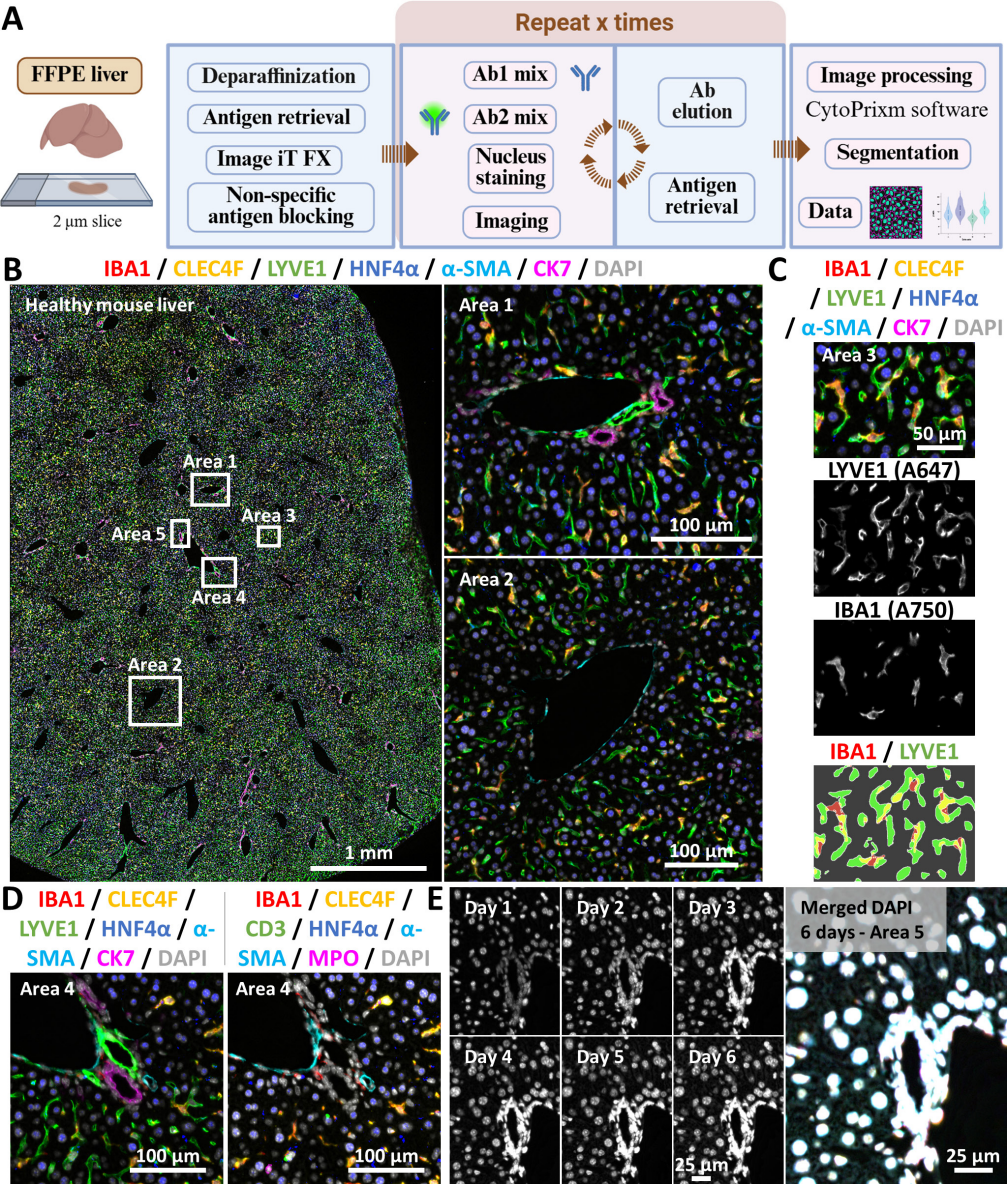
High-dimensional imaging of FFPE mouse liver tissues by mIF. (**A**) Sequential mIF simplified workflow to analyse FFPE liver samples. Panel A was created with BioRender.com. (**B**) An FFPE liver section from an adult healthy mouse was analysed using the mIF method. Typical output images after alignment are displayed. Areas of interest are delineated and shown in the following panels at a higher magnification. (**C**) Area 3 highlights a selected region of the liver and displays single channel as well as merged images and binary segmentation (lower image) of the two indicated markers. (**D**) Area 4 was used to demonstrate the possibility of using different sets of markers to highlight specific cellular contexts according to the researcher’s objectives. (**E**) Nuclear staining (DAPI) over six consecutive days of imaging and antibody stripping is shown individually and as a merged image for Area 5, as a quality control for tissue integrity. The following fluorophores were conjugated to the appropriate secondary antibodies: α-SMA: A488; CD3: A647; CK7: A750; CLEC4F: A647; HNF4α: A750; IBA1: A750; LYVE-1: A647; MPO: A555. Ab, antibody; DAPI, 4′,6-diamidino-2-phenylindole; FFPE, formalin-fixed paraffin-embedded; mIF, multiplex immunofluorescence.

We and our collaborators have widely used our previously published protocol for mIF for the analysis of liver sections.^25^,^27^ Figure 2A presents the simplified workflow to enable the analysis of FFPE tissue sections. Figure 2B–E display the final output after image acquisition and preprocessing in CytoPrixm and demonstrates the application of the technique to an FFPE liver section from an adult healthy mouse. As shown in figure 2B, tile scanning offers the opportunity to obtain a broad overview of the tissue architecture and cellular composition in a large representative area. To demonstrate the staining pattern in greater detail, specific regions were zoomed in. Area 1 corresponds to a portal tract containing branches of the portal vein, hepatic artery and bile duct, the latter visualised with an anti-CK7 antibody, while Area 2 corresponds to a pericentral space, identified by the presence of the central vein. Figure 2C shows Area 3, in which ECs (LYVE-1^+^) and macrophages (IBA1^+^) are shown with their very distinctive cell distribution and shapes. The lower image represents typical membrane overlaps between those two distinct cell types, which must be considered when performing high-end image analyses. To overcome this issue, in our laboratory, we first segment nuclei (DAPI) and then assign cell identity based on whether the nuclei are at least 60% overlapping with the respective membrane marker. This is important to prevent falsely concluding on the presence of biphenotypic cells; and one should assume it is not relevant to measure LYVE-1 staining intensity in IBA1^+^ macrophages. Notably, this approach does not fully resolve issues related to single cell phenotyping, for example, single cell-resolved lymphocyte expression of polarisation profile-specific markers in lymphoid structures with a high cell density and significant membrane contacts, for which specific algorithms must be developed depending on project-specific and antibody panel-specific considerations. Figure 2D shows Area 4, presenting two different sets of markers. All eight markers were stained on the same tissue slide and can be arranged and visualised according to the researcher’s objectives. The final panel (figure 2E), corresponding to the enlarged Area 5, illustrates DAPI staining over six consecutive days of mIF. Those DAPI acquisitions were merged into a single image, showing that the sample remains well preserved after multiple cycles, as evidenced by the overlapping signals in white.

**Figure 3:**
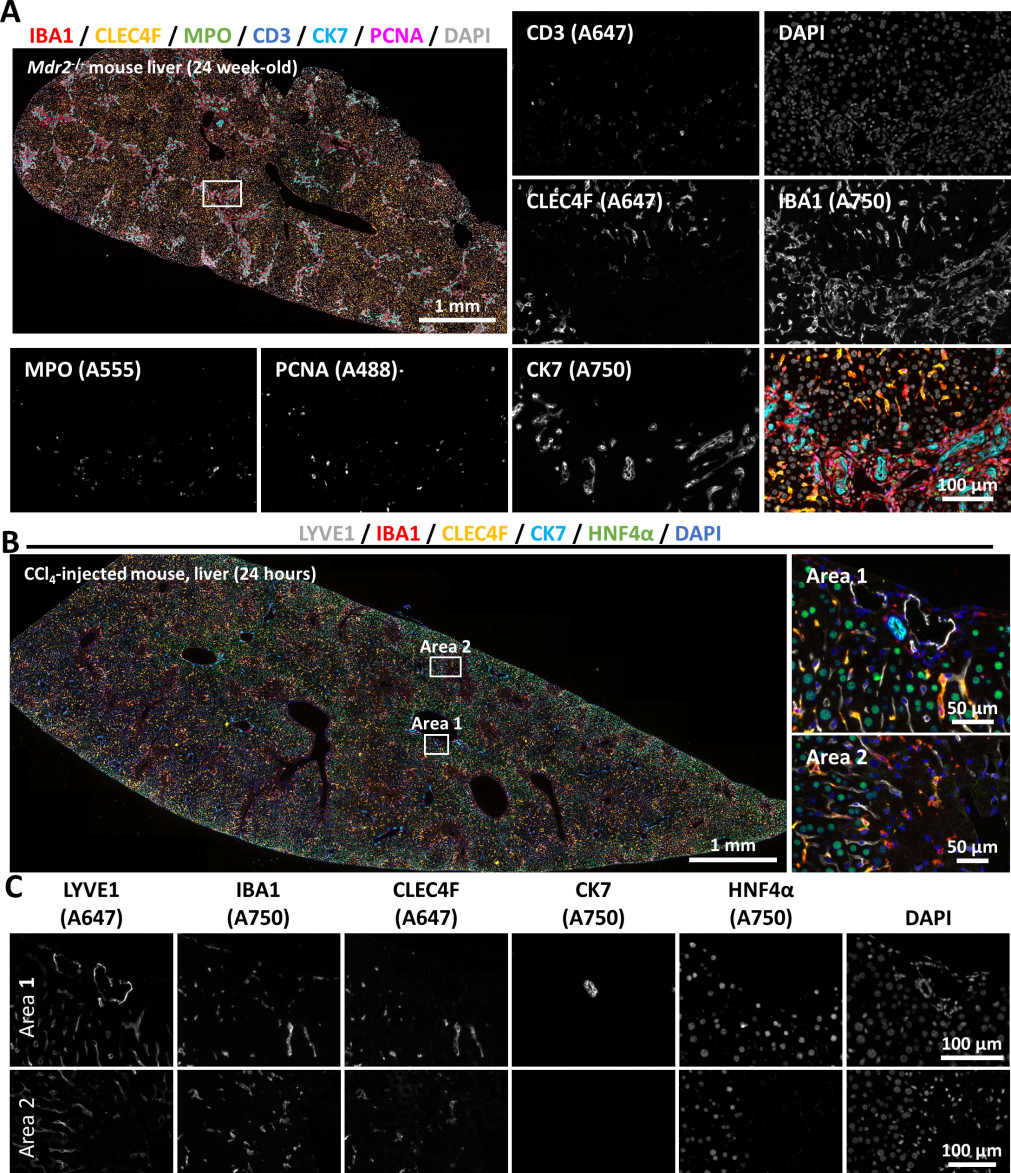
Sequential multiplex immunofluorescence reveals histological remodelling during liver injury. (**A**) An FFPE liver section from a mouse with chronic cholestatic disease (*Mdr2*^−/−^), showing inflammation-associated and tissue repair-associated markers displayed as single-channel or merged images. The white square indicates an enlarged region displayed in the smaller insets for improved visualisation. (**B**) Images obtained from an FFPE liver section from a mouse 24 hours after a single CCl_4_ injection. The white square highlights enlarged Areas 1 and 2 from the whole-slide scan, demonstrating the simultaneous comparison of seemingly unaltered (Area 1) versus necrotic (Area 2) tissue regions. (**C**) Single channel grayscale images from Areas 1 and 2 depicted in (**B**) are shown. The fluorophore conjugated to each secondary antibody used here is indicated in brackets on the single channel images. CCl_4_, carbon tetrachloride; DAPI, 4′,6-diamidino-2-phenylindole; FFPE, formalin-fixed paraffin-embedded; *Mdr2*, multidrug resistance 2.

Figure 3 shows the application of the technique to FFPE liver samples from mice with hepatic injuries. In figure 3A, the sample corresponds to a mouse liver with chronic cholestatic disease (*Mdr2*^−/−^ mice). In this model, reactive ductular cells expand along with extensive inflammation and fibrosis within the hepatic tissue.^28 29^ By combining immune cell markers (IBA1, CLEC4F, MPO and CD3), a biliary cell marker (CK7) and a proliferation marker (PCNA) into a single image, it becomes possible to accurately assess liver injury-related hallmarks and their interrelations on a wide tissue area. This example image further describes how a relatively simple protocol can be applied on a large scale for deciphering tissue changes along with phenotype progression. Similarly, figure 3B shows the liver of a mouse that received a single CCl_4_ injection to induce acute hepatic injury. In this model, it is possible to visualise uninjured portal areas (Area 1) or the border of a necrotic area (Area 2). The figure displays five markers for distinct cell populations; each stained in different cycles but on the same tissue section. Figure 3C shows the comparison between diseased (Area 2) and healthy areas (Area 1), stratified by singular markers.

By applying mIF in figures2  3, we observed specific tissue alterations that can be further explored in greater depth, according to the goals of the study, by incorporating additional disease-specific or cell-specific markers into the antibody panel. On the one hand, a growing number of studies now explore the use of highly-plexed methods for studying tissue landscapes. On the other hand, there is a crucial need for expanding the range of read-out methods applied to next-generation in vitro cell culture systems, a field that is currently developing rapidly.

### Application to in vitro models of liver diseases: FFPE intrahepatic cholangiocytes organoids

**Figure 4:**
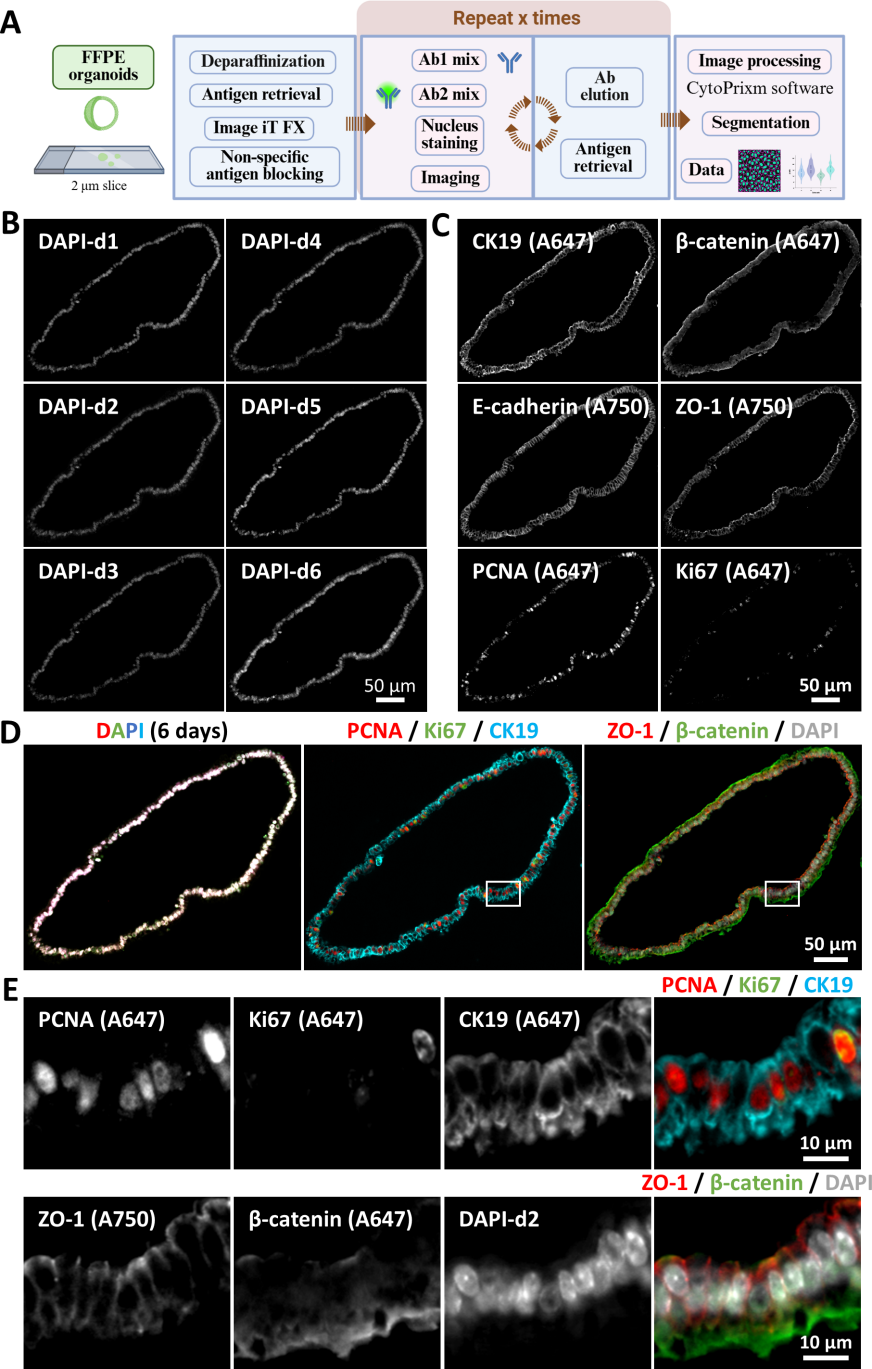
Dissecting liver organoid architecture by multiplex immunofluorescence. (**A**) Adapted sequential multiplex immunofluorescence workflow for the analysis of FFPE sections from mICOs. Panel A was created with BioRender.com. (**B**) Nuclear staining used over 6 consecutive days of multiplexing highlights sample integrity. (**C**) Single channel images show multiple markers that can be successfully acquired from a single mICO sample. (**D**) Merged channels demonstrate the combination of different markers, enabling the evaluation of specific characteristics in mICOs. The white square indicates the enlarged area shown in panel **E**. (**E**) Enlarged area showing single channel and merged images from **D**. The fluorophore conjugated to each secondary antibody used here is indicated in brackets on the single channel images. Ab, antibody; DAPI, 4′,6-diamidino-2-phenylindole; FFPE, formalin-fixed paraffin-embedded; mICOs, mouse intrahepatic cholangiocyte organoids.

The workflow shown in figure 4A highlights key adjustments made to our protocol for enabling mIF on FFPE sections from mouse intrahepatic cholangiocyte organoids. Since those samples were more prone to physical damage during longer incubations, antibody stripping was shorter. Figure 4B shows the DAPI nuclear staining for each staining cycle performed on the same slide, allowing assessment of sample integrity after five stripping cycles. Figure 4C displays the different markers used for characterising the cells that compose the organoids. Finally, in figure 4D, the overlay of DAPI from each staining cycle is shown, where whiter regions indicate nuclear overlap. In the same panel, the merged images of all markers are presented, enabling the evaluation of cell cycle-related protein expression (PCNA and Ki67) and cell–cell adhesion (ZO-1 and β-catenin) within the organoids. Additionally, images as shown in figure 4E allow us to monitor the polarisation that cholangiocytes maintain under standard culture conditions, with the apical surface facing the lumen and the basal surface oriented toward the exterior of the organoids.^30^

### Analysis of multiple markers at the single cell level in primary cell cultures

**Figure 5:**
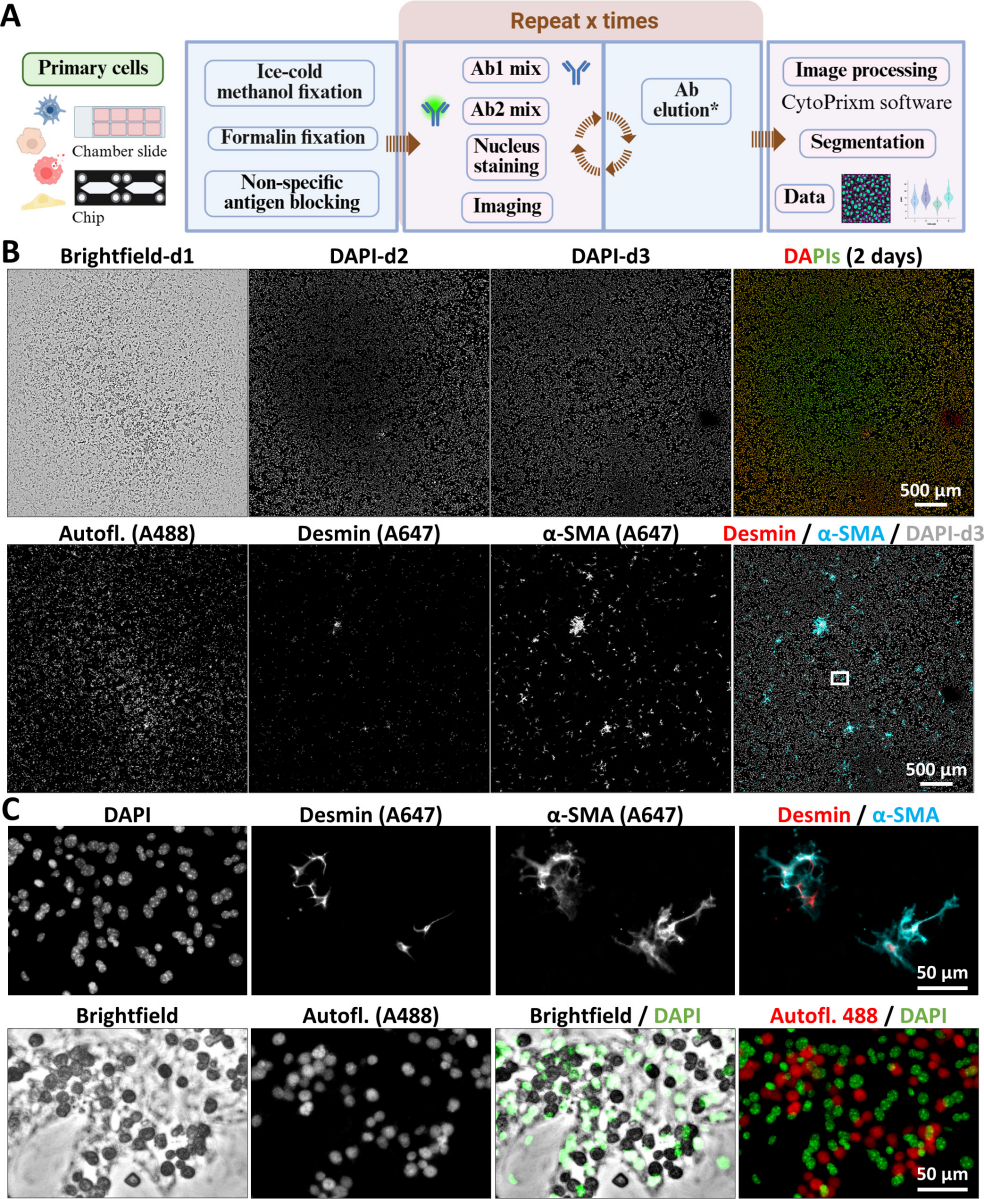
Multiplex immunofluorescence as a tool for quality assessment of primary liver cell cultures. (**A**) Sequential multiplex immunofluorescence workflow for the analysis of primary liver cells in culture. *A modified antibody‑elution method that omits the water‑bath step. Panel A was created with BioRender.com. (**B**) Primary mouse hepatocytes and hepatic stellate cells were isolated from a healthy mouse, then seeded. After adherence, cells were fixed in ice-cold methanol and paraformaldehyde. DAPI images demonstrate that cells remain intact after antibody stripping, while single-channel images confirm the specificity of each staining. A brightfield image was also acquired for gross evaluation of the cultures. The brightfield image shown here represents the final output after shading correction of individual tiles, image stitching, followed by Gaussian background correction using division in FIJI. (**C**) Enlarged areas highlighting the staining pattern and typical morphology of two hepatic stellate cell markers, as well as sample autofluorescence. The fluorophore conjugated to each secondary antibody used here is indicated in brackets on the single channel images. Ab, antibody; DAPI, 4′,6-diamidino-2-phenylindole.

While organoid-based research is crucial in studies needing a high throughput, primary cells freshly isolated from organs still represent a valuable model for in vitro mechanistic investigations.^31^ In figure 5A, we introduce a workflow for sequential mIF applied to primary liver cells in culture. We found that an initial fixation step with ice-cold methanol followed by a second fixation with PFA provides optimal conditions. Furthermore, the antigen-retrieval step can be omitted, making the protocol faster and gentler. Figure 5B shows both brightfield and fluorescence images of a chamber slide seeded with a mixture of primary mouse hepatocytes and HSCs. The DAPI-stained nuclei images demonstrate that the cells remain intact after stripping. In addition, as shown here, it is important to also acquire an image of the autofluorescence signal, which can later be subtracted during image processing as needed, to support relevant signal analyses. Here, we show some images obtained with markers specific for HSCs (desmin and α-SMA), which we deemed robust because these cells are easily recognised by their characteristic shape, and the signals were not overlapping with autofluorescence. Figure 5C shows zoomed-in images for a better assessment of the quality of staining. In the DAPI staining, nuclei of different sizes can be observed; the larger nuclei likely correspond to hepatocytes, whereas the smaller ones belong to other cell types. Using the two selected markers, HSCs can be clearly identified while the cells with larger nuclei are negative for desmin and α-SMA. As mentioned previously, autofluorescence, emitted primarily by dead cells and generally in the 488 and 555 channels, can be detected and considered without compromising quantitative analysis, as shown when autofluorescence and DAPI images are overlaid. The presence of cell debris is commonly observed in primary cell cultures. However, as shown in the next figures, those should ideally be washed out prior to experiment initiation.

**Figure 6:**
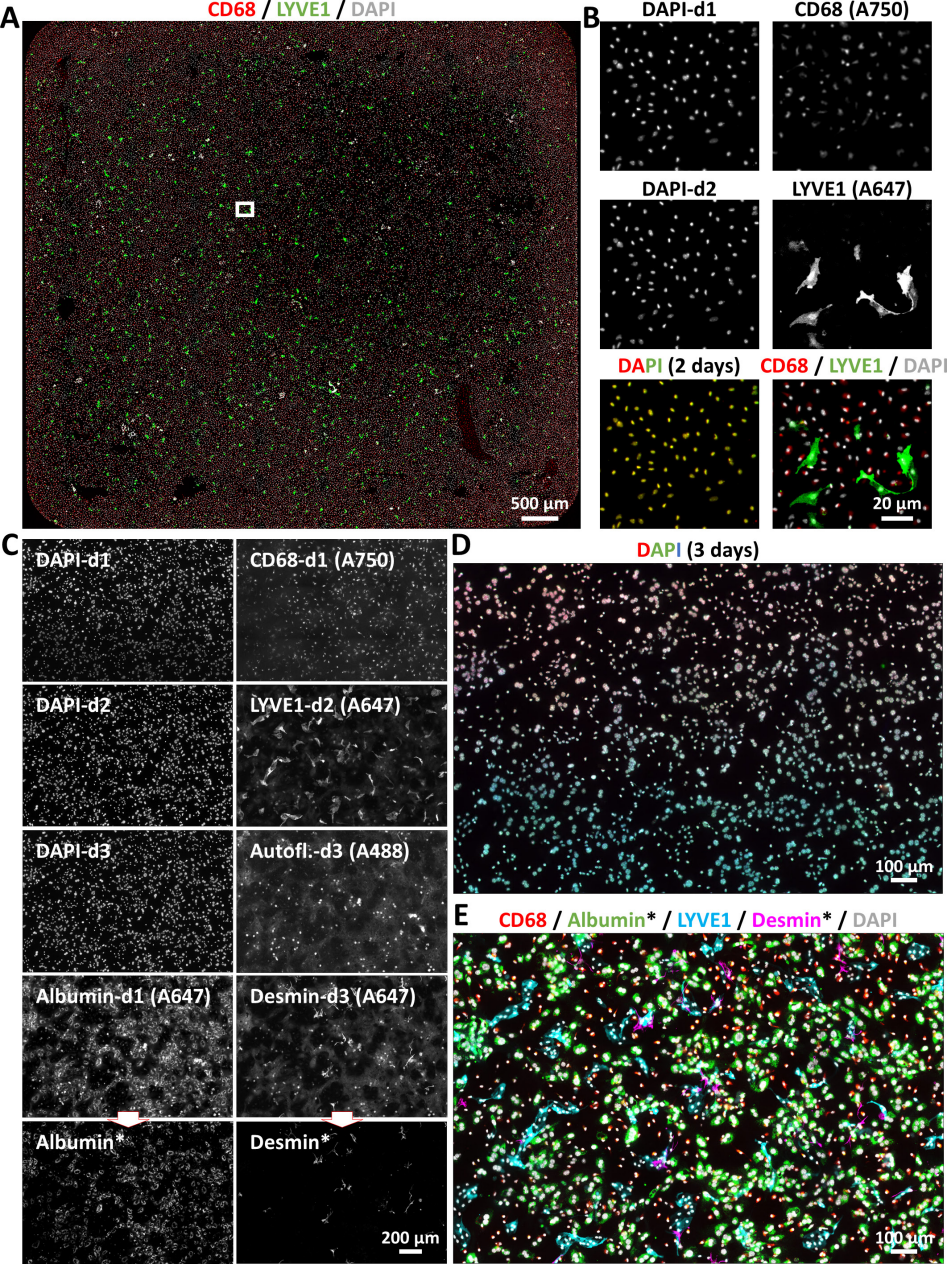
Cell identity and morphology characterisation in complex primary liver cell cultures assessed by mIF. (**A**) Our mIF workflow as described in figure 5A was applied to a mixed population of primary macrophages (CD68^+^) and ECs (LYVE-1^+^) isolated from a healthy mouse liver. (**B**) Enlarged images showing DAPI-stained nuclei before and after a stripping cycle, demonstrating that cells remain intact throughout mIF processing, along with single-channel and merged images from both days. (**C**) Three cycles of mIF were performed on a mixed population of primary mouse macrophages (CD68^+^), ECs (LYVE-1^+^), hepatic stellate cells (desmin^+^) and hepatocytes (albumin^+^), along with nuclear staining. The asterisk indicates images from which the autofluorescence image has been subtracted. (**D**) Merged nuclear staining images from three consecutive days of mIF. (**E**) Merged images illustrating the phenotypic characterisation of each mouse liver cell population cultivated in the same well. The fluorophore conjugated to each secondary antibody used here is indicated in brackets on the single channel images. DAPI, 4′,6-diamidino-2-phenylindole; ECs, endothelial cells; mIF, multiplex immunofluorescence.

To further validate the staining of different primary hepatic cell types, we seeded a mixture of primary mouse liver ECs and macrophages onto chamber slides. Figure 6A presents a whole-well view, while figure 6B shows a magnified region containing DAPI-stained nuclei of all cells on day 1 and day 2 of the experiment. The merged images demonstrate that no cell loss occurred during the procedure. The images on the right side of figure 6B show CD68^+^ cells (macrophages) from the first staining cycle, LYVE-1^+^ cells (ECs) from the second cycle, and a merged image combining both days.

To confirm the overall efficiency of the workflow, primary hepatocytes, macrophages, ECs and HSCs were coseeded in chamber slides. Figure 6C shows the results of three consecutive days of mIF. On day 1, CD68^+^ macrophages and albumin^+^ hepatocytes were detected. On day 2, LYVE-1^+^ ECs were visualised, and on day 3, desmin^+^ HSCs were identified. As previously noted, autofluorescence may interfere with signal detection; therefore, autofluorescence subtraction was applied for the albumin and desmin channels, and the resulting images are marked with an asterisk. Figure 6D shows a merged image of nuclear staining across the three cycles, and figure 6E presents the merged image of all labelled cell populations, confirming the success and reliability of the technique.

### Implementation of multiplex immunofluorescence in the study of next generation liver-on-a-chip models

**Figure 7:**
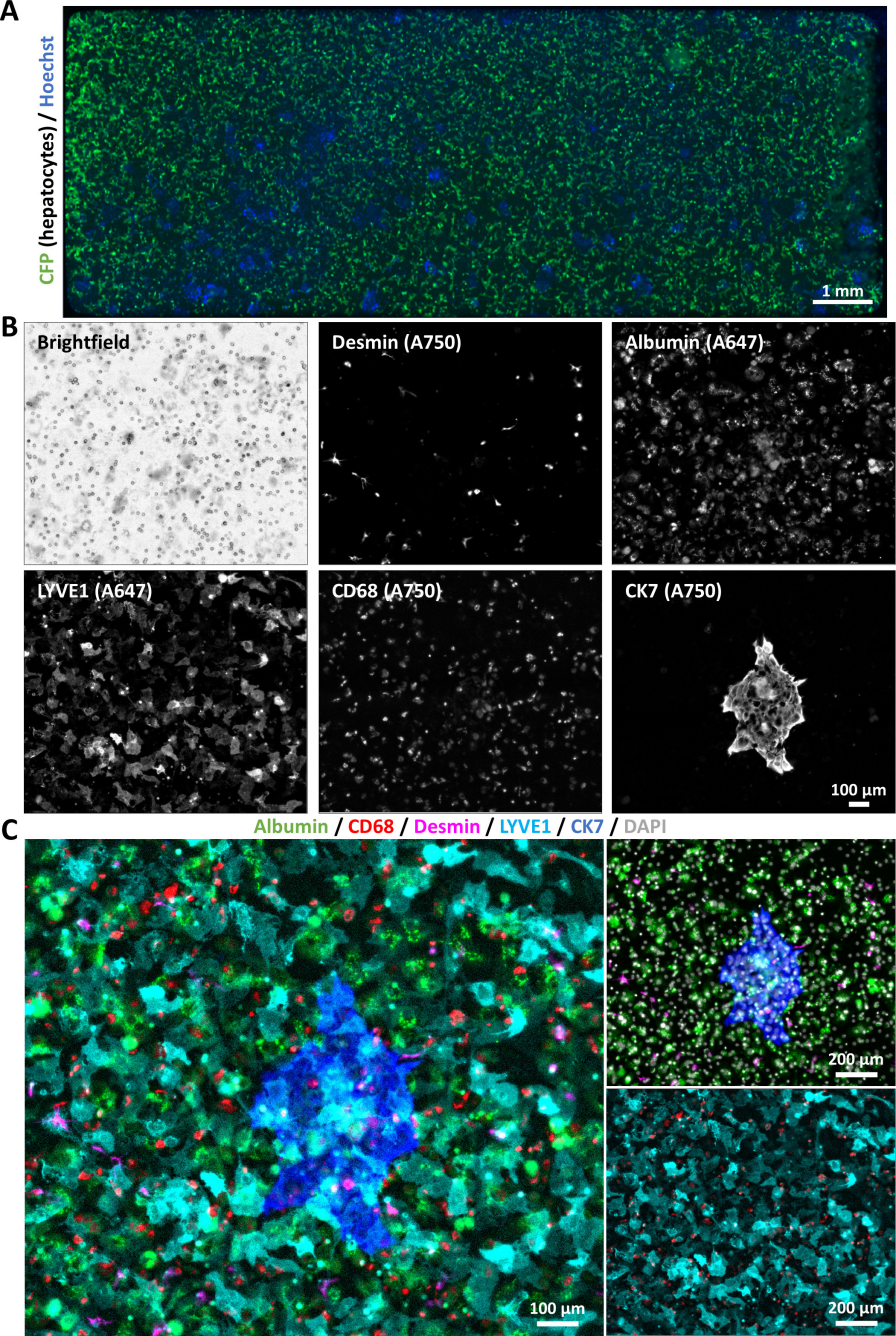
Next-generation biliary niche-on-a-chip models can be fully characterised using an antibody stripping-based mIF protocol. (**A**) Whole-scan image of a biochip membrane prior to cell fixation, showing cellular nuclei stained with Hoechst 33342 and hepatocytes derived from a reporter transgenic (actin-CFP) mouse. (**B**) Single-channel representations from sequential mIF performed in the biliary niche-on-a-chip, showing multiple cell populations: desmin^+^ cells represent hepatic stellate cells, albumin^+^ cells represent hepatocytes, LYVE-1^+^ cells represent ECs, CD68^+^ cells represent liver macrophages and CK7^+^ cells represent liver intrahepatic cholangiocytes. (**C**) Merged images derived from different markers acquired during sequential mIF within the same biochip, either with all or a subset of markers displayed as merged images. The fluorophore conjugated to each secondary antibody used here is indicated in brackets on the single channel images. DAPI, 4′,6-diamidino-2-phenylindole; ECs, endothelial cells; mIF, multiplex immunofluorescence.

The establishment of an mIF workflow for primary cells enabled the application of this methodology to complex in vitro systems, such as biochips. Figure 7A shows a general view of the biliary niche-on-a-chip BoC prior to cell fixation, established as previously described by our group.^29^ In this experiment, the biochip was seeded with hepatocytes derived from reporter transgenic (actin-CFP) mice, and the nuclei of the living cells were stained with Hoechst. Figure 7B includes a brightfield image of the biochip, showing both the cells and the 8 µm-wide pores of the membrane (small perfectly round objects) that separates the two cellular compartments, along with the fluorescence images corresponding to each labelled cell population (ie, desmin^+^ HSCs, albumin^+^ hepatocytes, LYVE-1^+^ ECs, CD68^+^ macrophages and CK7^+^ biliary epithelial cells). Finally, figure 7C shows the merged images, enabling the simultaneous visualisation of all cellular populations.

### Translational relevance for studying liver diseases in human samples

**Figure 8:**
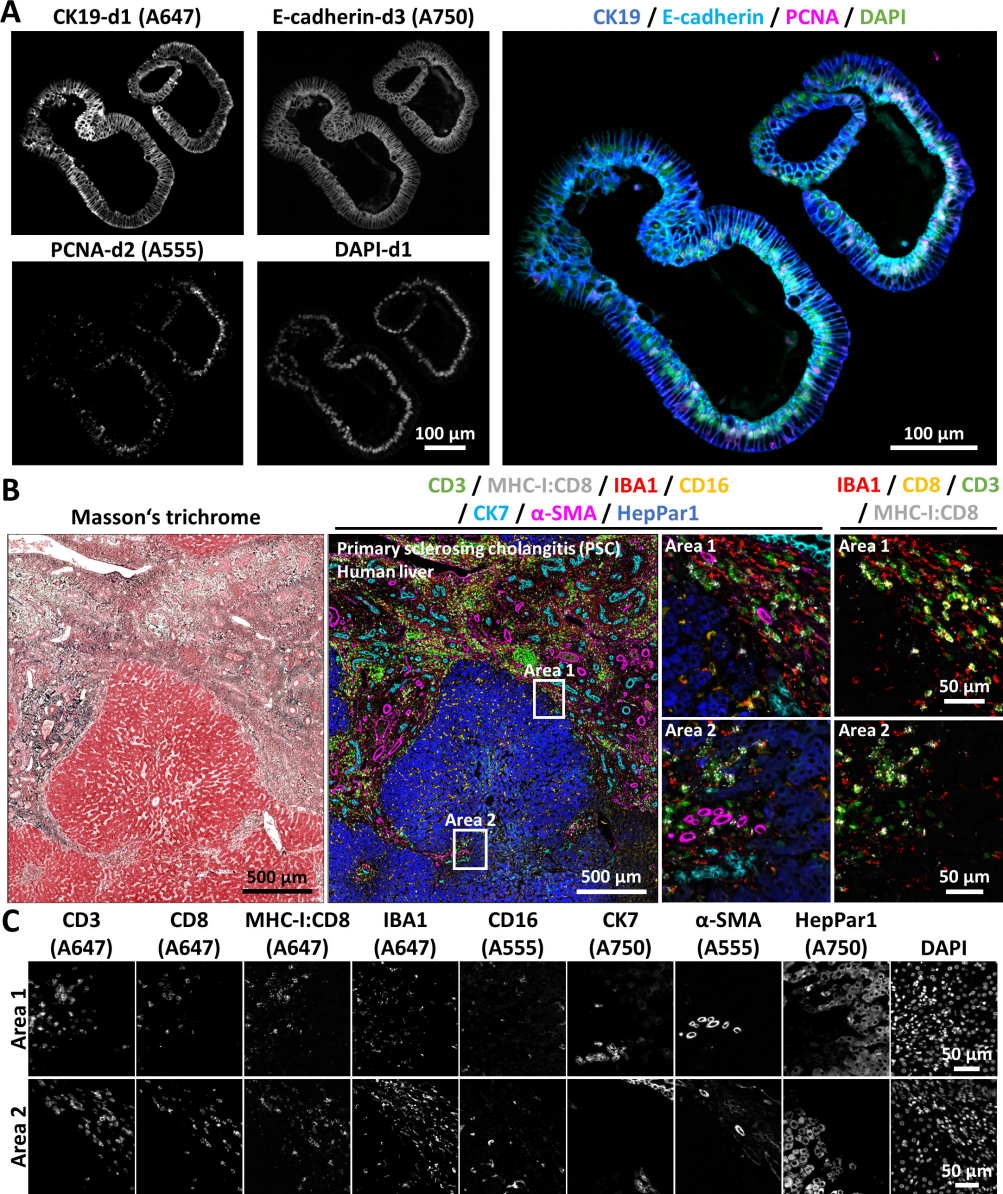
Enabling high-end phenotyping of patient-derived liver organoids and FFPE liver sections by using mIF and proximity ligation assays. (**A**) mIF applied to an FFPE section of hICOs, showing nuclear staining, marker-specific single-channel images and merged images. (**B**) An FFPE liver section from a human patient with PSC was retrieved. A large-field scan shows Masson’s trichrome staining and mIF output images. Areas 1 and 2 are shown at higher magnification to provide improved resolution of marker distribution patterns. (**C**) Marker-specific single-channel images from Areas 1 and 2 are shown for comparative analysis. The fluorophore conjugated to each secondary antibody used here is indicated in brackets on the single channel images. DAPI, 4′,6-diamidino-2-phenylindole; FFPE, formalin-fixed paraffin-embedded; hICOs, human intrahepatic cholangiocyte organoids; MHC, major histocompatibility complex; mIF, multiplex immunofluorescence.

Antibody stripping-based mIF offers many opportunities due to its versatility. Figure 8A illustrates the application of the technique to characterise organoids derived from human cholangiocytes, in which cells were labelled with anti-CK19, anti-PCNA and anti-E-cadherin antibodies across three consecutive cycles of mIF. Similarly, figure 8B demonstrates the analysis of a human FFPE liver section from a person with PSC, which underwent multiple cycles of immunofluorescence, simultaneously revealing the expression of eight proteins within the same sample along with a Masson’s trichrome counterstaining. In addition to the technology described in previous figures, we successfully integrated the use of a proximity ligation assay (PLA). PLA detects protein–protein interactions by amplifying a fluorescent signal, here for instance only when MHC-I and CD8 proteins are in proximity (<40 nm). This approach enables the visualisation of a functional aspect, that is, the engagement of cytotoxic CD8^+^ T cells with tissue cells. Importantly, this allows the researchers to generate stronger evidence of cell–cell interactions, as compared with the mere observations of cells being localised next to each other. As the PLA technology uses primary antibodies together with secondary oligonucleotide-coupled antibodies, it is applicable to other protein–protein interactions of interest, as long as suitable primary antibodies are available. Finally, figure 8C displays Areas 1 and 2 stratified by individual markers, illustrating the specificity of each staining, including the PLA.

## Discussion

In recent years, we have witnessed major and exciting technological breakthroughs that not only allowed the research community to largely expand the portfolio of experimental approaches but also increased the need for specialised reagents, equipment and personnel. Within this context, it appears crucial for all laboratories not to ‘miss the train’ of technological advances. Yet, simpler methods remain highly valuable, particularly for screening or for validation purposes.^32^ These considerations are especially relevant for understanding disease biology in relation to the spatial organisation of the cellular environment, which is the focus of the rapidly growing field of spatial biology. With this in mind, we optimised a protocol based on antibody stripping on archival FFPE sections^19 22^ for the study of liver diseases. In this study, we elaborate on new applications of this sequential mIF protocol, in vitro models such as two-dimensional primary cultures, organoids and liver-on-a-chip platforms. We deem this protocol simple to implement, and cost-efficient and time-efficient. Additionally, we introduce an open-source, in-house–designed software (CytoPrixm) that streamlines image processing after acquisition.

Since our first publication in 2020,^20^ our protocol has been further optimised, allowing its adaptation to innovative in vitro models beyond its original application to liver FFPE sections. Extending its use to complex experimental platforms, including systems that aim to recapitulate the functional architecture of the organ, may provide biologically relevant responses across diverse disease contexts. This is particularly timely given the rapid evolution of engineered in vitro models, which offer reliable and translatable alternatives that may complement or in some cases even replace traditional animal models.^33^ We have extensively used this technology in a growing number of studies across distinct experimental settings, demonstrating the method’s robustness, reproducibility and, most of all, versatility as reflected by its rapid implementation. For instance, we were able to dissect the KC niche and its implications in the neonate liver immunity^34^ and explore a patient sample from a previously uncharacterised syndrome.^35^ More recently, we applied the protocol described here in fetal liver-like organoids to evidence the multipotency of this new model of liver development and disease.^36^

Beyond visualisation, imaging techniques can be combined with machine-learning segmentation tools and high-end analyses, referred to as imaging cytometry. As such, our multiplex imaging workflow has proven to hold the potential of playing a fundamental role in studies led by our group or conducted through collaborations in a wide variety of settings. As a typical example, we were able to evidence on >100 liver sections from patients with MASLD, MASH, PSC, PBC and severe alcohol-related hepatitis and corresponding mouse models, the relevance of the spatially resolved macrophage and neutrophil phenotyping along with ductular reaction.^26 37^ In line, mIF revealed the intricate roles of monocytes in the repair of liver necrotic regions, through crosstalk pathways involving multiple cell types.^27^ We further used mIF for in-depth characterisation of histological changes in mouse models of liver injury to guide the design of a primary cell-based liver-on-a-chip model.^38^ In line, we evidenced crucial differences between hepatocytes and biliary epithelial cell-derived ductular cells in their response to liver injury, through distinct expression patterns of the acute phase protein orosomucoid 2 (ORM2).^29^ Beyond liver disease, we used mIF in studies related to protein carbamylation in atherosclerotic plaques,^39^ revealed the retinol saturase (RetSat) expression pattern in intestines,^40^ or monocyte migration promoting ferroptosis in lung epithelial cells.^41^

Reports of the use of mIF in primary cells are scarce in the literature and, when available, typically involve highly specialised approaches that rely on complex technologies. A recent study reported the scission-accelerated fluorophore exchange method, a spatiotemporal mIF imaging strategy capable of sequentially labelling multiple molecular targets within the same sample.^42^ This technique was developed to enable rapid labelling, cleavage-based removal and relabelling of fluorophores in living cells, allowing the visualisation of numerous targets over time without compromising cellular viability.^42^ In contrast, our methodology represents a simpler yet robust alternative that can be readily and reproducibly implemented in routine laboratory workflows.

It is important to highlight the existence of advanced platforms for spatial biology analysis and multiplexed phenotyping, such as imaging mass cytometry, which uses mass spectrometry combined with antibodies conjugated to heavy-metal isotopes^43^; MACSima, which is based on CycIF with antibodies conjugated to fluorophores that are subsequently deactivated^44^; and the PhenoCycler (formerly known as CODEX), which employs CycIF relying on antibodies tagged with DNA barcodes and complementary fluorescent reporter probes.^45^ These technologies offer very high levels of multiplexing, often exceeding 60 markers, and remain extremely valuable in various experimental and exploratory contexts. However, they require substantial financial investment and rely on predefined panels, which may limit their implementation in some settings. Recent transcriptome-based spatial analysis platforms (eg, 10x Xenium) also provide subcellular resolution while measuring a large number of markers in a standardised way. However, the detection limits for individual genes are often high, and the dynamic range favours strongly expressed transcripts. Our approach, in contrast, allows for fine-tuning of detection limits and dynamic range for each marker individually, which is particularly important for lowly expressed proteins like cytokines and transcription factors, or for the visualisation of very rare cell populations.

In addition, we here also provide CytoPrixm, an open-source software we developed to consolidate image processing steps into a single interface and without the need for prior knowledge in coding languages. This image preparation tool allows the experimenter to circumvent an otherwise time-consuming stage that is particularly prone to human errors. However, it is expected that such tools may soon be replaced by artificial intelligence-based assistants that may perform similar tasks. Significant advances in data analysis are likely to occur in the near future, although the role of critical scientific judgements should not be underestimated.^32^

### Study limitations

This study has several methodological limitations. The immunofluorescence technique requires non-negligible amount of hands-on time, as it is not automated and requires substantial effort for both image acquisition and subsequent data processing. In addition, depending on the specimen used and the study aims, the protocol will potentially generate large imaging datasets that require careful preprocessing prior to quantitative analysis and necessitate appropriate computing resources. During image analysis, potential artefacts, such as partial membrane overlap between distinct but neighbouring cell populations or autofluorescence, must also be considered in a project-specific and antibody panel-specific manner to avoid misinterpretation of the results. At the experimental level, the efficiency of antibody stripping must also be carefully verified using consistent imaging parameters to ensure complete signal removal before subsequent staining cycles. Furthermore, some antibodies may lose functionality after repeated stripping cycles, underscoring the importance of preserving epitope integrity to maintain effective antigen–antibody recognition. Finally, the integrity of tissue samples is critical for obtaining reliable results. Indeed, archival sections that may not have been stored optimally may not sustain a high (>5) number of staining cycles and require repetition of the experimental workflow into distinct antibody panel batches followed by careful imaging data integration afterwards. Notably, the immunofluorescence methodology described herein was originally tailored to FFPE tissues and was not suitable for unfixed samples (eg, fresh frozen tissue sections that do not sustain the chemical antibody stripping procedure). The present work further expanded the applications to primary and organoid cell cultures, as well as our liver-on-a-chip platform. The CytoPrixm software here provided is aimed at facilitating image processing after the immunostaining approach has been completed, and preparing for in-depth image analyses. The latter functions are not currently included in our software solution and still require substantial, project-specific considerations and trained personnel.

## Conclusions

The spatially resolved and multidimensional in situ characterisation of single cells within complex systems, whether in their native tissue or in advanced cell culture models, opens a broad range of opportunities in the era of omics analyses. Such approaches may enable in-depth mechanistic investigations and research model qualification, increase research reproducibility, support diagnostic approaches and guide therapeutic decision-making, among many other applications. We here provide an A-to-Z toolbox for mIF on the most common sample types making the core of scientific projects nowadays. Notably, all protocols described in this study rely on off-the-shelf reagents and a conventional fluorescence microscope, together with open-source software, making the overall workflow widely accessible. By providing an overview of possible applications and future expandability, we empower researchers across diverse fields to broaden the applications of this technique, offering unparalleled flexibility and adaptability throughout the experimental process.

### Novelty

Here, we introduce a fully integrated, end-to-end workflow leveraging open-source tools, which enables mIF across tissue, organoids and primary cell systems in a highly time-efficient and resource-efficient manner. This approach delivers a broadly versatile, accessible and scalable solution for translational research in hepatology.

## Supplementary material

10.1136/egastro-2026-100379 online supplemental file 1

10.1136/egastro-2026-100379 online supplemental file 2

10.1136/egastro-2026-100379 online supplemental file 3

## Data Availability

Data are available upon reasonable request.
