# Supplementary material for: Unified platform for multiplex immunofluorescence across liver tissues and engineered models
Source: eGastroenterology. 2026 Apr 30;4(2):e100379. doi: 10.1136/egastro-2026-100379 (PMC13141115; doi:10.1136/egastro-2026-100379)
Supplement: online supplemental file 1 [file egastro-4-2-s001.pdf]

Supplementary Methods

Data processing workflow in CytoPrixm

Raw .czi files containing unstitched tile scans or single-scene acquisitions, are imported, validated, and processed to extract imaging metadata. Large-area scans are reconstructed into stitched TIFF images using Fiji-based stitching. Prior to stitching, individual tiles are shadow corrected using a per-slice shading subtraction approach. Tile images are assembled using the Grid/Collection Stitching plugin in Fiji.[1] Tile positions are defined explicitly using a TileConfiguration.txt file, ensuring that spatial coordinates and tile ordering follow the acquisition geometry and are not inferred by feature-based alignment. This approach guarantees reproducible image geometry and is consistent with microscope-provided tile layouts. Overlapping regions are fused using linear blending, which provides smooth intensity transitions while preserving relative signal intensities established during shadow correction. Subpixel accuracy is enabled to refine tile alignment beyond integer pixel resolution, and overlap computation is explicitly activated to ensure correct blending weights in overlapping areas. Alignment refinement is constrained using conservative displacement thresholds (regression threshold = 0.10, maximum-to-average displacement ratio = 2.50, absolute displacement threshold = 3.50 pixels) to prevent unstable local corrections and preserve global mosaic geometry. Tiles are processed at full spatial resolution without down sampling, and no pre-alignment downscaling of images is performed. To optimize performance for large, high-resolution datasets, computation is configured to prioritize speed at the expense of increased memory usage. The full mosaic bounding box is computed, and the complete stitched image is generated independently of the current viewport. Intermediate XML outputs are disabled, and the final stitched mosaic is written directly to disk.

Final bit-depth conversion (e.g., 8-bit export) preserving original intensity is applied only after stitched image generation, ensuring that stitching and blending are performed on linearly corrected, full-precision image data. Individual fluorescence channels (typically 3–4 per acquisition date) are renamed, evaluated, and carried forward for alignment. Because the region of interest often shifts across staining cycles, images must be spatially registered. The pipeline performs channel alignment using extracted metadata. Image alignment is performed in Fiji using Register Virtual Stack Slices, which internally relies on SIFT-based feature matching. Among available Fiji registration tools, Register Virtual Stack Slices and HyperStackReg were evaluated, with the former selected due to its robustness for large, multi-channel images acquired across staining cycles. Feature extraction is based on the Scale Invariant

Feature Transform (SIFT) framework. Interest points are detected using a Difference-of-Gaussian approach to ensure scale invariance. Correspondences between images are identified by matching local feature descriptors and filtered using Random Sample Consensus (RANSAC) to remove outliers. The resulting landmark sets are used to compute spatial transformations for image registration. For the Scale Invariant Interest Point Detector, the following parameters are used: an initial Gaussian blur of σ_0 = 1.6 px, 12 steps per scale octave, a minimum image size of 64 px, and a maximum image size of 1024 px. All other detector parameters ae left at their default values. Feature matching employes a SIFT feature descriptor with a descriptor size of 8 and 8 orientation bins per 4×4 px block, as recommended for robust matching of large-scale biological images.[2] Geometric consistency of matched features is enforced using a geometric consensus filter with a minimal inlier ratio of 0.05, allowing transformations to be accepted even when only a small fraction of matches represented true correspondences.[2] All remaining parameters are kept at default settings. For registration, a rigid transformation model (translation and rotation) is applied. Images are registered into a common coordinate space defined by a reference image, selected as the image with the largest spatial dimensions. Shrinkage constraints are disabled, and all other parameters of Register Virtual Stack Slices are used with default values. Following alignment, the pipeline performs tissue-focused cropping (manual, semiautomatic, or automatic), background subtraction, and channel merging. Background subtraction is performed in Fiji using the Rolling Ball Background Subtraction algorithm.[3] A rolling ball radius of 50 pixels is used as default setting. This radius removes low-frequency background variations while preserving cellular and subcellular signal structures. All other parameters are kept at their default values, and background subtraction is applied independently to each channel prior to channel merging. DAPI images are segmented using the adapted deep-learning model implemented in the cellsegpackage, generating nuclear masks for downstream quantitative analyses.

CytoPrixm software architecture

All major components were implemented in Python, selected for its scientific ecosystem and compatibility with machine learning frameworks. Deep-learning–based segmentation is executed using TensorFlow. Environment and dependency management are handled via conda-pack packages to ensure reproducibility across systems. ImageJ/Fiji tools are integrated through the im-jy-package, written in Jython and packaged as a single Maven-built *.jar* file.

CytoPrixm output and usability

The pipeline logs all processing steps, warnings, and errors to a dedicated log file and organizes outputs into a structured directory hierarchy. A graphical user interface enables users to configure parameters, execute individual steps, and process large datasets with minimal manual intervention.

Counterstaining of liver sections

Masson's trichrome was used as a counterstain to visualize collagenous connective tissue fibers in human liver sections after the whole sequential immunostaining was completed, according to the manufacturer's instructions (Abcam, Cambridge, UK).

Imaging

For image acquisition, slides were mounted with VectaMount AQ Aqueous Mounting Medium (Vector Laboratories, Newark, CA, USA), covered with coverslips shortly (2-3 minutes) before imaging on a Zeiss Axio Observer 7 microscope (Carl Zeiss, Oberkochen, Germany) equipped with a Plan-Apochromat 20×/0.8 Ph2 M27 (dry) objective and a ZEISS Axiocam 712 mono camera. Alexa Fluor® 488, Alexa Fluor® 555, Alexa Fluor® 647, and Alexa Fluor® 750 were used as fluorophores. After imaging, slides were immediately immersed in distilled water until the coverslip detached and were then subjected to the next staining cycle, starting with the antibody stripping step. In the case of chamber slides and biochips, the cells remained submerged in distilled water throughout all steps, without coverslips. Images were acquired in the same sample regions through the consecutive days of the procedure.

Primary mouse liver cell isolation and culture

Following euthanasia by isoflurane overdose, distinct hepatic cell populations (hepatocytes, HSCs, KCs, and ECs) were isolated from fresh liver tissue of C57BL/6J wild-type (WT) or reporter transgenic (actin-CFP) mice after digestion with type IV collagenase, according to previously described protocols.[4] Hepatocytes were separated using a 45% Percoll gradient, while HSCs were isolated using a 33.3% Nycodenz gradient. Magnetic bead–based separation

with F4/80$^+$ or CD146$^+$ antibodies and MACS columns (Miltenyi Biotec) was applied to purify KCs and ECs, respectively, following the manufacturer's instructions. After isolation, cells were cultured in William's E medium containing 10% fetal bovine serum and 1% antibiotics, either in monoculture or in co-culture, on collagen-coated µ-Slide 8 Well chamber slides (ibidi GmbH, Gräfelfing, Germany). Chamber slides were maintained in a humidified incubator at 37°C with 5% CO_2 for 24 to 72 hours prior to fixation.

Mouse liver-on-a-chip seeding and culture

The liver biochip was assembled following the protocol described by our group with some modifications.[4, 5] In brief, freshly isolated mouse liver cells were prepared following the protocol described above. To obtain BECs, *Mdr2*$^{-/-}$ organoids were thawed, expanded in Matrigel, and later released using Cultrex Organoid Harvesting Solution. During dissociation, organoids were intently preserved as cell aggregates instead of being fully separated into single cells with gentle dissociation. To prepare the biochip, each cell type was independently isolated, combined in the desired proportions, and seeded onto the sterile, collagen-coated chip (Dynamic42, Jena, Germany). The biochips have an upper chamber, a lower chamber, afferent and efferent channels, and an integrated porous membrane separating the chambers. The parenchymal (hepatocytes, HSCs and BECs) and vascular (KCs and ECs) layers were seeded on consecutive days in William's E medium containing 10% fetal bovine serum and 1% antibiotics. The liver biochip platform was maintained in a humidified incubator at 37°C with 5% CO_2. The following day, the chip was perfused with circulating immune cells isolated from mouse blood, for 30 minutes using a peristaltic pump at a speed of 400 µL/min.

Intrahepatic cholangiocyte organoid generation

Mouse intrahepatic cholangiocyte organoids (mICOs) were generated and characterized as previously described.[5, 6] Briefly, the livers of wild-type (WT) or *Mdr2*$^{-/-}$ mice were perfused and digested. Primary biliary epithelial cells (BECs) were isolated using magnetic-activated cell sorting (MACS) with mouse EpCAM antibody-conjugated beads. BECs were seeded into Matrigel and cultured in mouse liver isolation medium [6] for 3 to 4 days, after which the medium was replaced with mouse liver expansion medium. mICOs were then generated within 7 to 14 days and cultured in Matrigel. For organoid analyses, mICOs were incubated in Cultrex organoid harvesting solution (Bio-Techne GmbH, Germany) for 30 minutes at 4 °C to remove Matrigel, followed by two washes with cold PBS. Organoids were then fixed in 4% PFA for 1

hour at room temperature and washed twice with cold PBS. Next, organoids were dehydrated and stained in 0.5% eosin dissolved in 96% ethanol, embedded in paraffin, and sectioned for immunostaining. The organoids were not centrifuged to prevent alterations but instead let to settle under gravity. Alternatively, mICOs were dissociated using Cultrex organoid harvesting solution for generating single cell suspensions of biliary cells, prior to seeding onto collagen-coated chamber slides or biochips.

Supplementary references

1. Preibisch S, Saalfeld S, Tomancak P. Globally optimal stitching of tiled 3D microscopic image acquisitions. Bioinformatics. 2009;25(11):1463–5.
2. Lowe DG. Distinctive Image Features from Scale-Invariant Keypoints. International Journal of Computer Vision. 2004;60:91–110.
3. Sternberg S. Biomedical Image Processing. Computer. 1983;16(1):22–34.
4. Liu H, Yin G, Kohlhepp MS, et al. Dissecting Acute Drug-Induced Hepatotoxicity and Therapeutic Responses of Steatotic Liver Disease Using Primary Mouse Liver and Blood Cells in a Liver-On-A-Chip Model. Adv Sci (Weinh). 2024;11(30):e2403516.
5. Liu H, Yin G, Franco Leonardi B, et al. Reactive cholangiocyte-derived ORM2 drives a pathogenic modulation of the injured biliary niche through macrophage reprogramming. Gut. 2025;74(10):1694–710.
6. Broutier L, Andersson-Rolf A, Hindley CJ, et al. Culture and establishment of self-renewing human and mouse adult liver and pancreas 3D organoids and their genetic manipulation. Nat Protoc. 2016;11(9):1724–43.
