# Supplementary material for: Unified platform for multiplex immunofluorescence across liver tissues and engineered models
Source: eGastroenterology. 2026 Apr 30;4(2):e100379. doi: 10.1136/egastro-2026-100379 (PMC13141115; doi:10.1136/egastro-2026-100379)
Supplement: online supplemental file 2 [file egastro-4-2-s002.docx]

**Supplementary Table 1. Primary antibodies**

| Antigen | Manufacturer | Catalog Nº. | Clone | Host species |
| --- | --- | --- | --- | --- |
| Albumin | Novus Biological | NBP1-32458 | polyclonal | Rabbit |
| CD16 | Abcam | ab183354 | SP175 | Rabbit |
| CD3 | Cell Signaling | 78588S | E4T1B | Rabbit |
| CD3 | Agilent | M725429-2 | F7.2.38 | Mouse |
| CD68 | Abcam | ab53444 | FA11 | Rat |
| CD8 | Biozol | ODN-DIA-TC8 | TC8 | Mouse |
| CK7 | Abcam | ab181598 | EPR17078 | Rabbit |
| CLEC4F | R&D Systems | MAB2784 | 370901 | Rat |
| Desmin | Abcam | ab15200 | polyclonal | Rabbit |
| E-cadherin | Cell Signaling | 3195 | 24E10 | Rabbit |
| HepPar1 | Agilent | M7158 | OCH1E5 | Mouse |
| HNF4α | Thermo Fisher | MA1-199 | K9218 | Mouse |
| IBA1 | VWR | 100369-764 | polyclonal | Rabbit |
| IBA1 | Abcam | ab283346 | EPR16589 | Rat |
| Ki67 | Abcam | ab16667 | SP6 | Rabbit |
| LYVE1 | Abcam | ab281587 | RM1008 | Rabbit |
| MPO | Abcam | ab208670 | EPR20257 | Rabbit |
| PCNA | Abcam | ab29 | PC10 | Mouse |
| ZO-1 | Abcam | ab221547 | EPR19945-296 | Rabbit |
| α-SMA | Abcam | ab124964 | EPR5368 | Rabbit |
| α-SMA | Agilent | M085129-2 | 1A4 | Mouse |
| β-catenin | Abclonal | A20221 | AMC0440 | Mouse |

**Supplementary Table 2. Secondary antibodies**

| Antigen, Fluorochrome | Manufacturer | Catalog Nº. | Host species |
| --- | --- | --- | --- |
| Mouse IgG, Alexa Fluor 555 | Cell Signaling | 4409S | Goat |
| Mouse IgG, Alexa Fluor 488 | Cell Signaling | 4408S | Goat |
| Mouse IgG, Alexa Fluor 750 | Abcam | ab175741 | Goat |
| Rabbit IgG, Alexa Fluor 647 | Cell Signaling | 4414S | Goat |
| Rabbit IgG, Alexa Fluor 750 | ThermoFisher | A-21039 | Goat |
| Rabbit IgG, Alexa Fluor 555 | Cell Signaling | 4413S | Goat |
| Rat IgG, Alexa Fluor 647 | Cell Signaling | 4418S | Goat |
| Rat IgG, Alexa Fluor 750 | Abcam | ab175751 | Goat |

**Supplementary Table 3:** **CytoPrixm Modules and Licences**

| Component | Key libs | Licence |
| --- | --- | --- |
| Stitching | ImageJ, Bio-Formats | Public Domain/BSD-2 + GPL (Bio-Formats readers may be GPL) |
| Datacheck GUI | PySimpleGUI | Custom freeware/commercial licence |
| Alignment | Register_Virtual_Stack_MT | GPL-3 |
| Background Adjustment | ImageJ core | PD/BSD-2 |
| Cropping | ImageJ core / Pillow / NumPy | PD/BSD/MIT |
| DAPI Prep | OpenCV, NumPy, Pillow | Apache-2 + BSD + MIT |
| DAPI Segmentation | TensorFlow, NumPy, tifffile, CellSeg (MIT) | Apache-2 + BSD + MIT |
| DAPI Post-Processing | OpenCV, skimage, Pillow | Apache-2 + BSD + MIT |
| Hyperstack Generation | ImageJ core | PD/BSD-2 |
| Channel Merge | ImageJ core | PD/BSD-2 |
| Image Size Correction | ImageJ core | PD/BSD-2 |
| Results Output | Python stdlib | PSF |
| GUI | tkinter | PSF + Tcl/Tk |
| main.py / framework | pandas | BSD-3 |
| macro.py | Java runtime | Oracle/OpenJDK |
| setup_logger | Python stdlib | PSF |

Supplementary Table 3 summarizes the software components used throughout the pipeline and their corresponding licence types. All major dependencies originate from widely adopted open-source ecosystems such as Python, Fiji/ImageJ, and TensorFlow, and rely on permissive or academically compatible licences (like PSF, MIT, BSD, Apache-2.0). These licences are standard in scientific software and allow open redistribution, reuse, and modification. They do not impose restrictive obligations on derived work, which makes CytoPrixm suitable for open-source release.

**Supplementary Table 4: Imaging metadata for the sample dataset used in this comparison.**This imaging dataset corresponds to a 155-tile scan which was acquired using a Plan-Apochromat 20x/0.8 M27 objective.

| Cycle # | Exposure time #1 DAPI (ms) | Exposure time #2 AF488 (ms) | Exposure time #3 AF647 (ms) | Exposure time #4 AF750 (ms) |
| --- | --- | --- | --- | --- |
| 1 | 2.5 | 24 | 150 | 250 |
| 2 | 3.0 | 24 | 180 | 300 |
| 3 |  |  | 100 |  |
| 4 |  |  | 42 |  |
| 5 |  |  | 60 |  |
| 6 |  |  | 60 |  |
| 7 |  |  | 60 |  |
| 8 |  |  | 30 |  |

**Supplementary Table 5: Hardware requirements and recommendations for running CytoPrixm on large-scale images.**

| **CPU requirement for CytoPrixm** | Multi-core CPU recommended; minimum 8 cores, practical 16+ cores |
| --- | --- |
| **RAM requirement** | Minimum 32 GB, practical 64–128 GB for large datasets |
| **GPU requirement** | Optional for general preprocessing; recommended for DAPI segmentation |
| **Recommended GPU** | NVIDIA GPU with at least 8–12 GB VRAM; practical 16+ GB for large batches |
| **Disk storage requirement** | High; typically several hundred GB to multiple TB per project |
| **Disk speed** | SSD strongly recommended |
| **Runtime scaling with tissue area** | linear |
| **Runtime scaling with number of image tiles** | linear |
| **Runtime scaling with number of staining cycles** | linear |
| **Alignment cost scaling** | Increases with image size and number of cycles |
| **Segmentation cost scaling** | Strong increase with total pixel count |

**Supplementary Table 6: Comparison of CytoPrixm processing times using two computers, on a large imaging dataset.**We used two computers: The PC was configured with an Intel i7-8565U CPU (4 cores, 8 threads) and 16 GB of RAM; no GPU was specified. The laboratory workstation was equipped with an Intel Xeon Gold 6244 CPU (8 cores, 16 threads, ~3.6 GHz), 192 GB of RAM, a 27.29 TB RAID array and a 477 GB SSD for storage, and an NVIDIA Quadro RTX 5000 GPU with 16 GB of memory.

|  | **PC** | **Lab workstation** |
| --- | --- | --- |
| **Image preparation** | 5 h 51 min | 3 h 05 min 59 s |
| **Datacheck** | 1 min 55 s | 2 min 3s |
| **Alignment** | 47 min 47 s | 1 h 48 min 35 s |
| **Crop (automatic)** | 3 min 54 s | 7 min 26 s |
| **Adjust background** | 56 min 34 s | 44 min 05 s |
| **Merged channels** | 24 min 59 s | 41 min 01 s |
| **DAPI segmentation** | 13 hours | 1 h 15 min 8 s |
| **Clean output** | 12 min 54 s | 23 min 17 s |
