# Supplementary material for: Unified platform for multiplex immunofluorescence across liver tissues and engineered models
Source: eGastroenterology. 2026 Apr 30;4(2):e100379. doi: 10.1136/egastro-2026-100379 (PMC13141115; doi:10.1136/egastro-2026-100379)
Supplement: online supplemental file 3 [file egastro-4-2-s003.pdf]

Unwatch 2

0 stars 0 forks 2 watching Branches Activity
Tags

Private repository

4 Branches 0 Tags Go to file t Go to file Add file Code

nkon887 add link to zenodo website c4d57e2 · 5 minutes ago

Multiplex_Pipeline_Execution	fix: adjust logger to store logs.log i...	4 months ago
Multiplex_package	fix: adjust readme due to updates i...	2 months ago
colab_scripts	feat: add colab notebook for the te...	3 years ago
ijm-macros	fix: exchange the macro_4_Merge_C...	4 years ago
im-jy-package	fix: adjust readme due to updates i...	2 months ago
.gitattributes	adding model for cellseg	3 years ago
.gitignore	feat: creating a python package dir ...	3 years ago
README.md	add link to zenodo website	5 minutes ago

README

Multiplex Fluorescence Immunostaining Analysis Pipeline For Preprocessing Of Czi Images

Pipeline Scheme

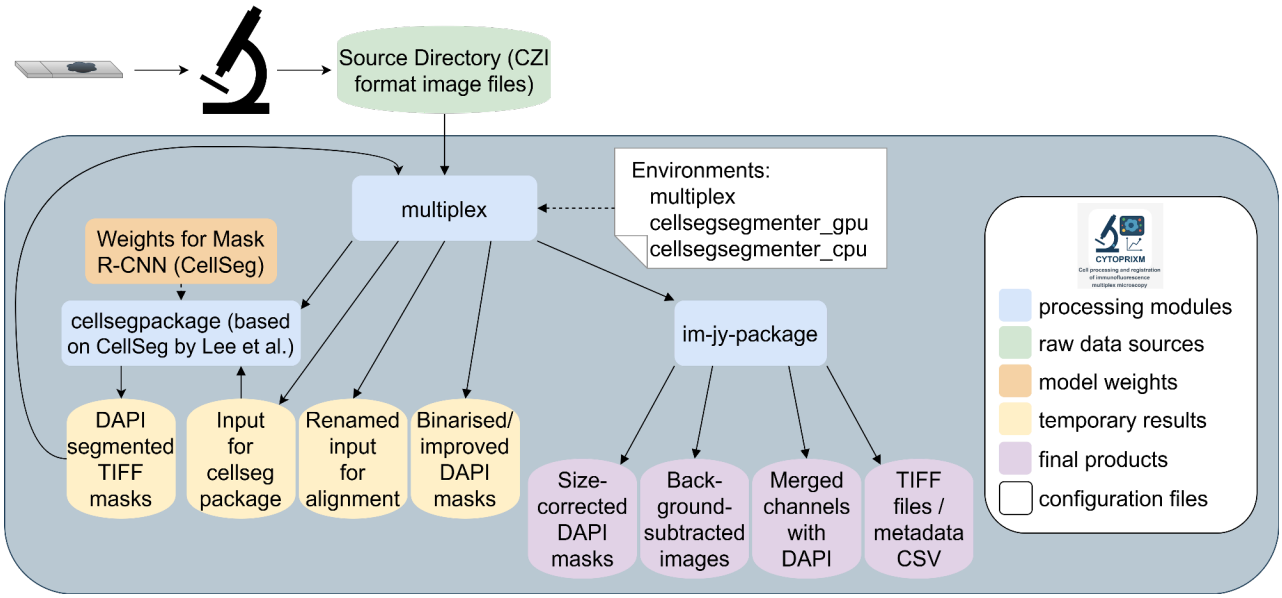

Description

This pipeline enables the user to prepare multiplex staining microscopy images for further tissue or cell population analysis. It consists of three main packages 1/ multiplex (python package), 2/ im-jy-package (Fiji package) and 3/ adapted cellsegpackage (python package using scripts and model from `https://github.com/michaellee1/CellSeg`) It converts czi unstitched data to stitched tiff images and extracts thereby necessary metadata. The czi files may consist of one scene (unstitched set of tile images with one or more channels or entire image sets (no tiles) containing channels) or series of scenes mixed with not image files. It works further with the tiff images and uses extracted metadata.

3, 4 channel images per date corresponding to one experiment are treated individually. Since the position of the region of interest changes from date to date, the channel images must be aligned. If the alignment of the raw data does not work properly, the images of each date are treated separately as a batch and processed (cropped) to repeat the alignment. It is possible to process more than one batch in one pass. After this process, the images are processed so that they are ready for further marker-specific segmentation and image analysis in other software.

The steps performed by this pipeline are:
large scan stitching > channel renaming > sequential image alignment > background subtraction and channel merging > DAPI segmentation
This pipeline generates all images required for: marker segmentation, imaging data generation and analysis

Software Requirements

1. Miniforge (Python >= 3.12, https://github.com/conda-forge/miniforge/releases/latest/download/Miniforge3-Windows-x86_64.exe)
2. Fiji (ImageJ 2.14.0/1.54f, Java 1.8.0_322, Jython, https://imagej.net/software/fiji/downloads)
3. Space for environments 6,56 GB

```
nvidia-smi
```

See example outpput below

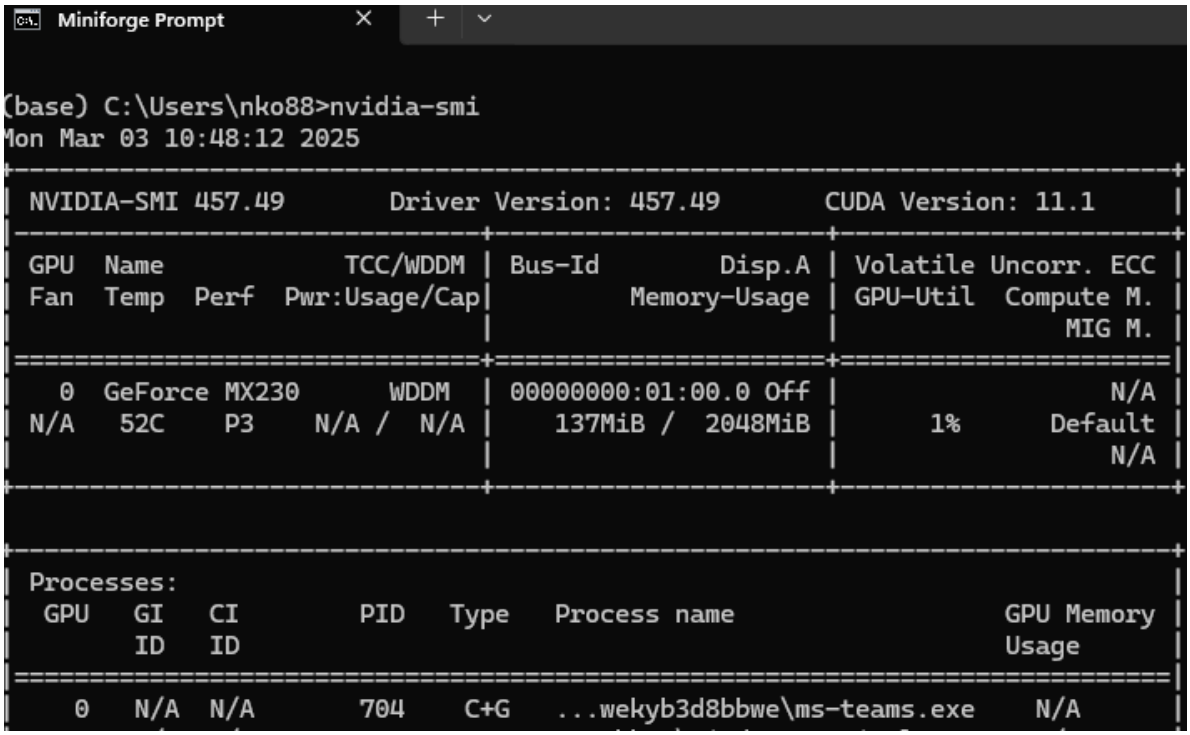

Supported Input Data

> **Caution**
>
> 1. Images as czi files without any `space` such as ` ` in the file name.
> 2. Each image (incl. shading correction if available) name should start with 6 digits stating the image acquisition date (e.g. 250701). The files should be named according to the scheme date(6 digits) underscore `_` patientID (replace the underscore `_` or the space ` ` within the patient ID with another character such as `-`) Example: 123456_patientID
> 3. The pipeline creates folders that the user should not change
> 4. Each batch should contain at least two channels(markers): DAPI and one another marker

Set-Up

1. Installation
    - Download the zipped file `pipeline.zip` from `Charite OneDrive` https://charitede-my.sharepoint.com/:f:/r/personal/natalja_amiridze_charite_de/Documents/Natalia/pipeline?csf=1&web=1&e=M9oMsL or from `Zenodo` https://doi.org/10.5281/zenodo.19059527. There are only `README.pdf` file, the folder `Multiplex_Pipeline_Execution` with the installation file `install.py`, the start software file `run_pipeline.py` and the folder im-jy-package with the file `im-jy-package-0.1.0-SNAPSHOT.jar` in it and the `tar.gz` files in the subfolder `tar_envs`. You must unzip the file `pipeline.zip` (once when you run the software for the first time). Skip this step next time
    - You can download and unzip this file https://drive.google.com/file/d/1wEOFDxaJG8FUGbCUdPaWrTWI1-_b1irC/view?usp=drive_link or from `Zenodo` https://doi.org/10.5281/zenodo.19059527 as test data. There you will find two czi files with a shading file and the description text file `StainingSequence.txt` with marker information
    - Install Miniforge and Fiji on your PC (once during first software execution). Next time skip this step
    - Set up the `FIJIPATH` environment variable (only once). Go to `Start` - `Edit system variables` - `Environment variables` or `Start` - `Edit environment variables for your account` - `Environment`

`variables`. There set the system variable `Variable name` to `FIJIPATH` and `Variable value` to the file location of ImageJ-win64.exe of your `Fiji` (once during first software execution). Next time skip this step

- Navigate to the execution folder `Multiplex_Pipeline_Execution` and run the commands in the Miniforge Prompt:

```
cd [...path-to-the-unzipped-downloaded-pipeline-
folder...]/Multiplex_Pipeline_Execution/
python install.py
```

If it is the first time, you're using the software and the environments are not already set, then you must wait until the environments are set (usually takes 5-10 minutes). Otherwise, you get the confirmation that the environments are set. Next time you don't have to install

2. Software Execution Run the following commands in the Miniforge Prompt:

```
  cd [...path-to-the-unzipped-downloaded-pipeline-
folder...]/Multiplex_Pipeline_Execution/
  python run_pipeline.py
```

After launching the pipeline, the graphical user interface (GUI) will appear—typically within a few seconds, but in some cases it may take several minutes (up to ~5 minutes). Please be aware that startup times can vary, and you may need to wait patiently for the interface to fully initialize (see Notes below). At the top is a bar with the menus `File`, `View`, and `Help`. Under `File` you get options to `Clear (Ctrl+L)` / `Copy (Ctrl+C)` / `Save (Ctrl+S) Log` of output window or to `Exit (Ctrl + Q)`. Under `File`, you will find options for `Delete (Ctrl+L)` / `Copy (Ctrl+C)` / `Save (Ctrl+S)` the Log of the `OUTPUT MESSAGES` window, or to `Exit (Ctrl+Q)`. Under `View`, you will find options for `Increase (Ctrl++/Ctrl+=)` / `Decrease (Ctrl+-)` / `Reset (Ctrl+0)` the font size. Under the `Help` menu, you will find the options `Help (F1)`, which opens a PDF file with a readme file, `Cite As (Ctrl + Shift + C)`, which displays a window with citation details, `Contact Us (Ctrl + Shift + U)`, which displays a window with author information, and `Set Recipient Email`, which displays a window, in which you must enter your email address. On the left side, you will find the `CytoPrixm` pipeline logo and buttons for executing the steps.

In the main interface window, you are asked to set `INPUT/OUTPUT PATHS` of source data and where to store output of the pipeline. Below are `STEP PARAMETERS`, which you have to set: like if you have a graphics processing unit (`GPU`) on your computer or not to select if you don't. It is important that you make your selection before you perform the `DAPISEG` step. The `Force Save` enables the user to overwrite the output data, which maybe already be present as output. The cropping options are to select a mode of the image cropping (`Crop mode`), depending on what you want. If you want to select image frame on your own, select then `Manual`, if you want to have already preselected frame, which excludes black area by creating a preselected rectangle frame around tissue, then `Semiautomatic` mode has to be selected and the user has then a possibility to check it and make final frame selection, how image should be cropped. Another mode `Automatic` is the mode where automatically the black area, which surrounds the issue will be automatically cropped out without user corrections. If you would like to be notified by email, select the `Notify By Email` option (if you have entered your email address in the top bar under `Help` – `Set Recipient Mail`, you should receive an email after completing the step you have performed). When you navigate to `STEP PARAMETERS` with the mouse, a screen tip appears. Below this is a window with `OUTPUT MESSAGES`, which displays all text outputs such as info, warnings, and errors. At the bottom left, you will find the status bar, which shows the current status of the pipeline and displays tips that change dynamically when you navigate with the mouse to the respective button or to the `INPUT/OUTPUT PATHS` setting.

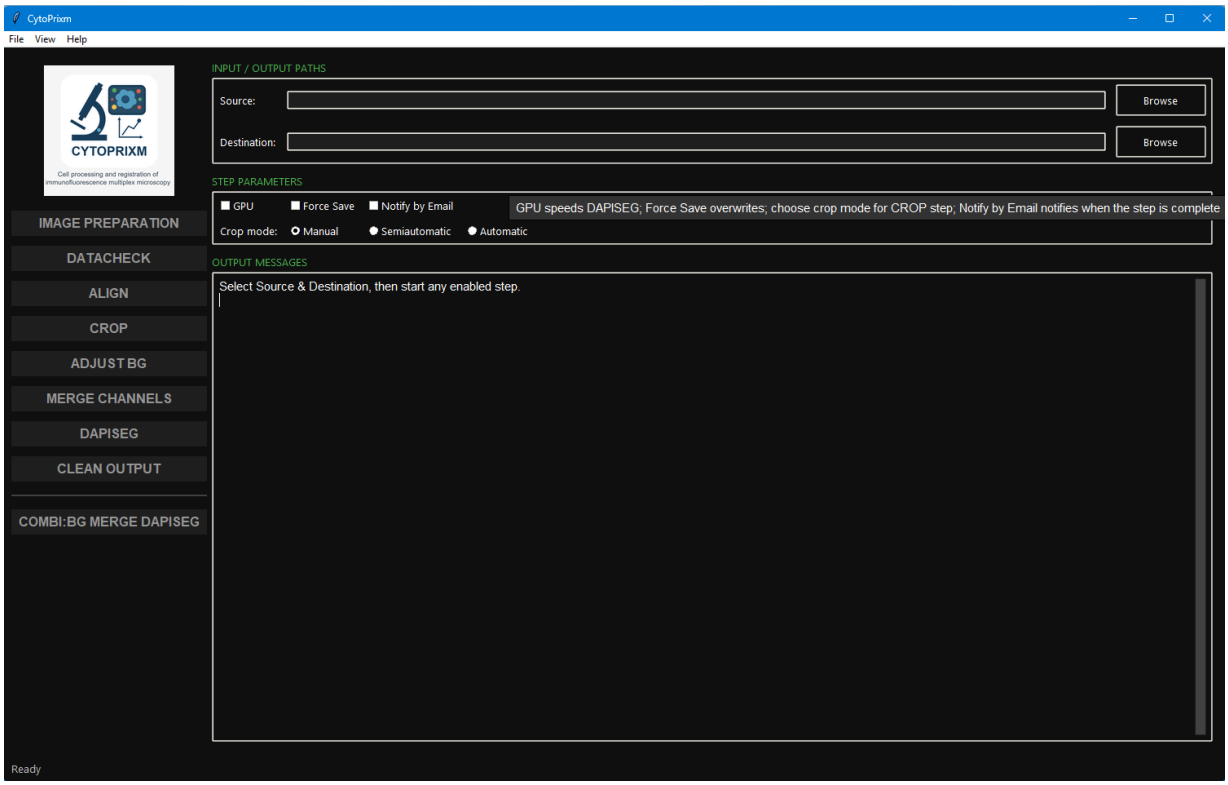

All buttons are deactivated on the left. In order to activate, you need to select the input location (where your raw czi data is located, usually in the microscopy-core server) and the destination location (where you want to store the output of the pipeline, recommended is local hard disk of a workstation if space is available. The path should not have any spaces in the names of the subfolders). The steps for which you provided input will be activated (if your target directory does not contain workingDir and subfolders, only the first step `IMAGE PREPARATION` will be activated. Otherwise, you can continue where you stopped with the next step of the pipeline or run the previous steps again). To execute the steps, you need to click the step button on the left side of the GUI window. All buttons are disabled during execution. In addition, you can see the dynamically changing state of the processed step in more detail in the main window under `OUTPUT MESSAGES` and by moving the process bar at the bottom right back and forth during execution. When one of the pipeline steps is complete, the button turns green and the next pipeline step button on the left is activated. This disappears when the process is complete. If you selected the `Notify By Email` option (if you have entered your email address in the top bar under `Help` – `Set Recipient Mail`, you should receive an email after completing the step you have performed. If your internet connection is restricted by the firewall (like in Charite), you may not receive the mail)

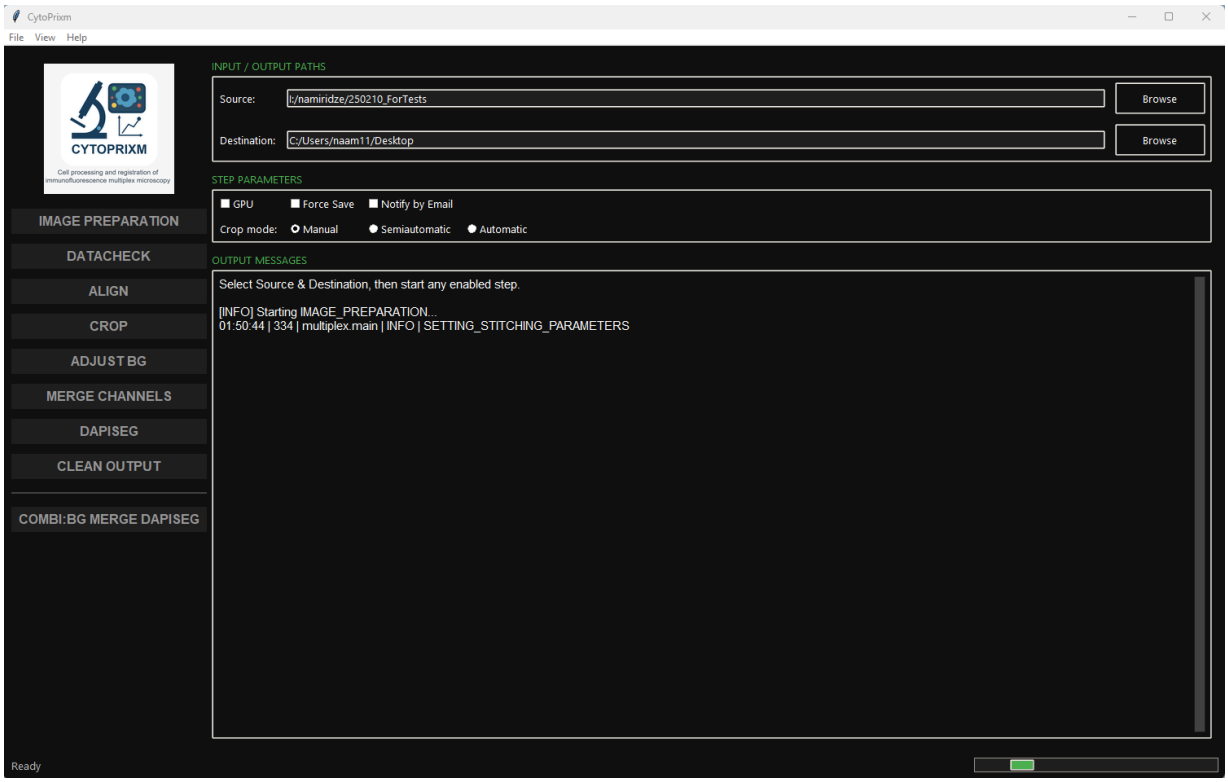

3. The structure for the processed image files in your destination directory is then:

```
     workingDir/
               /01_input
                        /date_caseID_subfolder(s)
               /02_alignment
                        /aligned stack(s) as tiff_file(s)
               /02_01_input_to_precrop
                        /date_caseID_subfolder(s) (if not missaligned copied from
01_input)
               /03_bg_processed
                        /caseID_subfolder(s) with background processed tiff file(s)
               /04_mergedChannels
               /05_dapi_seg
                        /01_input_folder
                        /02_seg_output
                        /03_dapi_seg_binary
                        /04_binary_size_correct
               /06_results_output
                        /CaseID subfolder(s) (nessesary output of all previous steps
combined for each CaseID)
                        /mergedChannels  containing CaseID subfolders with output tiff
images from the step
                         mergedChannels
               metadata.csv
```

Steps

1.

> **Caution**
>
> For the `IMAGE PREPARATION` step (im-jy-package) if you want shading correction, you must provide the shading correction file for each date with a name that includes the date (6 numbers like "230701") followed by the underscore `_` and word `shading` in czi format.

If you do not have the shading file, the `IMAGE PREPARATION` step can be done without the shading correction. During this step a dialog will appear where you have a list of selected files and not selected (the files that have names or format that don't follow the scheme), the user has to select the shading correction file (or no shading `No_shading_file`) for each date and resolution of output tif file (`8-bit` per default). Example:

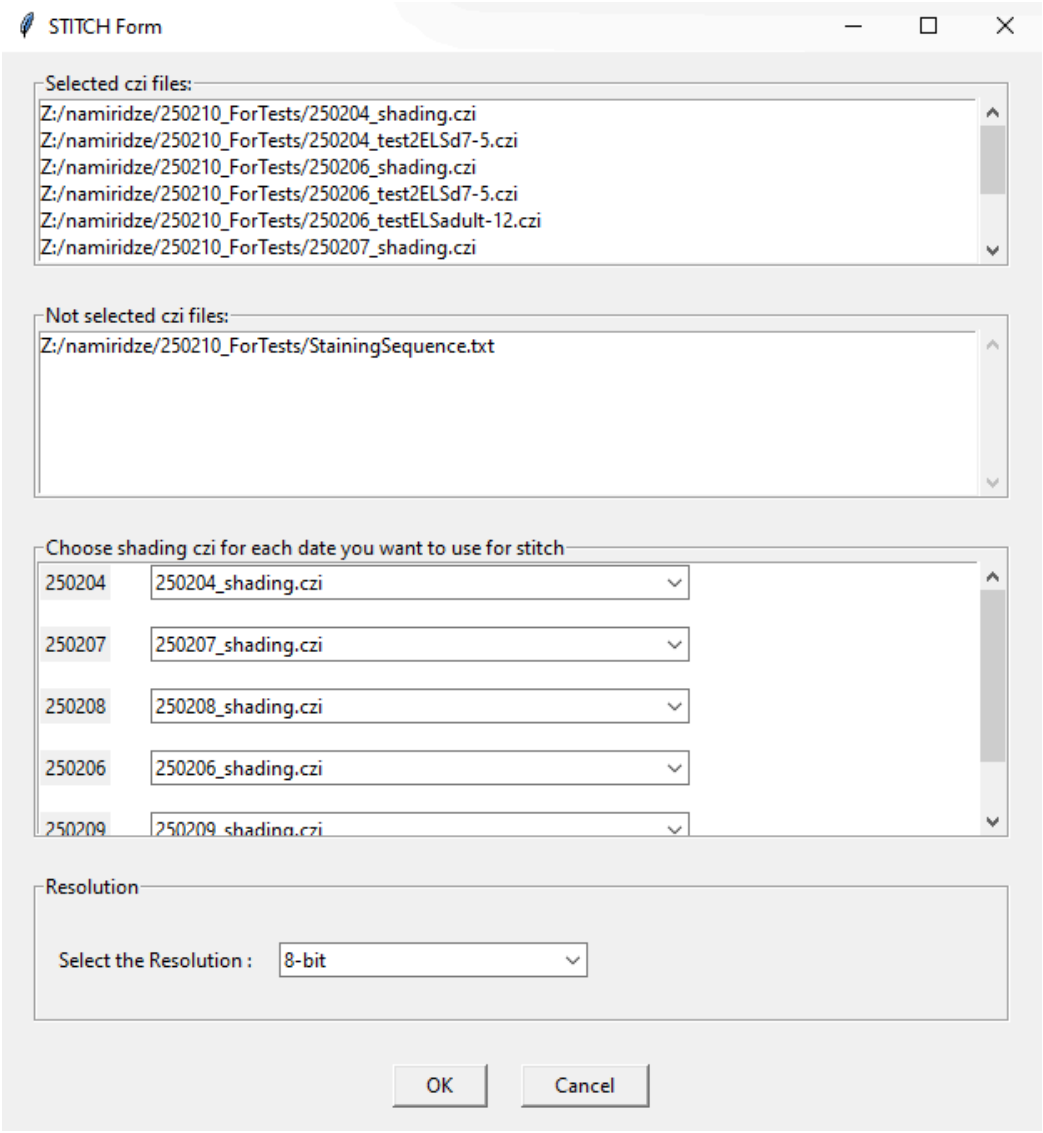

After submitting (clicking `OK`, `Cancel` ends the step), the `IMAGE PREPARATION` step is performed where Fiji will open and will either process the data or exit if you canceled the dialog. The images for each channel used are created and saved as tiff files in the date_sampleID subfolder in the `01_input` folder. Example:

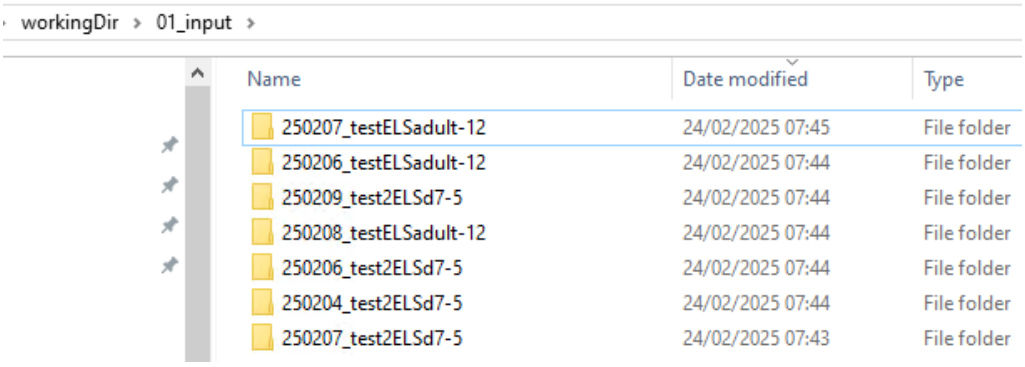

Also, the `metadata.csv` file is created in the workingDir. This csv file contains `date`, `expID`, `channel number`, `exposure time` for each channel, `ObjectiveModel`, `ObjectiveNominalMagnification`, `Default Channel #num` for each default channel for each processed czi file. Also columns `marker for channel #ChannelNumber` for corresponding channel will be stored and updated in the next step `DATACHECK`. Also, the sizes of each tif tile is stored in this file as `SizeX`, `SizeY`. Example:

date	expID	channel	channel	channel	channel	channel	channel	channel	channel	channel	channel	channel	channel	Exposure	Exposure	Exposure	Exposure	Exposure	Exposure	Exposure	Exposure	Exposure	Exposure	Objective	Objective	Default	Default	Default	Default	Default	Default	Default	Default	Default	Default	DefaultChannel	#11
220630	ROI-1	DAPI	AF647	AF750										150			3.578			700					Plan-A	20	AF647	Cy7	AF555	DAPI	EGFP	Bright	AF750	AF751	AF488	Cy3	Cy5	
220630	ROI-2	DAPI	AF647	AF750										150			3.578			700					Plan-A	20	AF647	Cy7	AF555	DAPI	EGFP	Bright	AF750	AF751	AF488	Cy3	Cy5	
220908	E3756(	DAPI	AF647	AF750										43.59			5.979			304.1					Plan-A	20	AF647	Cy7	AF555	DAPI	EGFP	Bright	AF750	AF751	AF488	Cy3	Cy5	
220909	E3756(	DAPI	AF647	AF750										59.6			5.37			161.9					Plan-A	20	AF647	Cy7	AF555	DAPI	EGFP	Bright	AF750	AF751	AF488	Cy3	Cy5	

When `IMAGE PREPARATION` is finished the button turns green and the next button `DATACHECK` is activated, and also you get on the right output in the `OUTPUT MESSAGES`. ALTERNATIVELY if you have more images that are to be added to the analysis: Run all previous steps from a distinct folder. After, copy/paste the content of the metadata file into the metadata from the first cycle. Copy the newly generated images into the input folder.

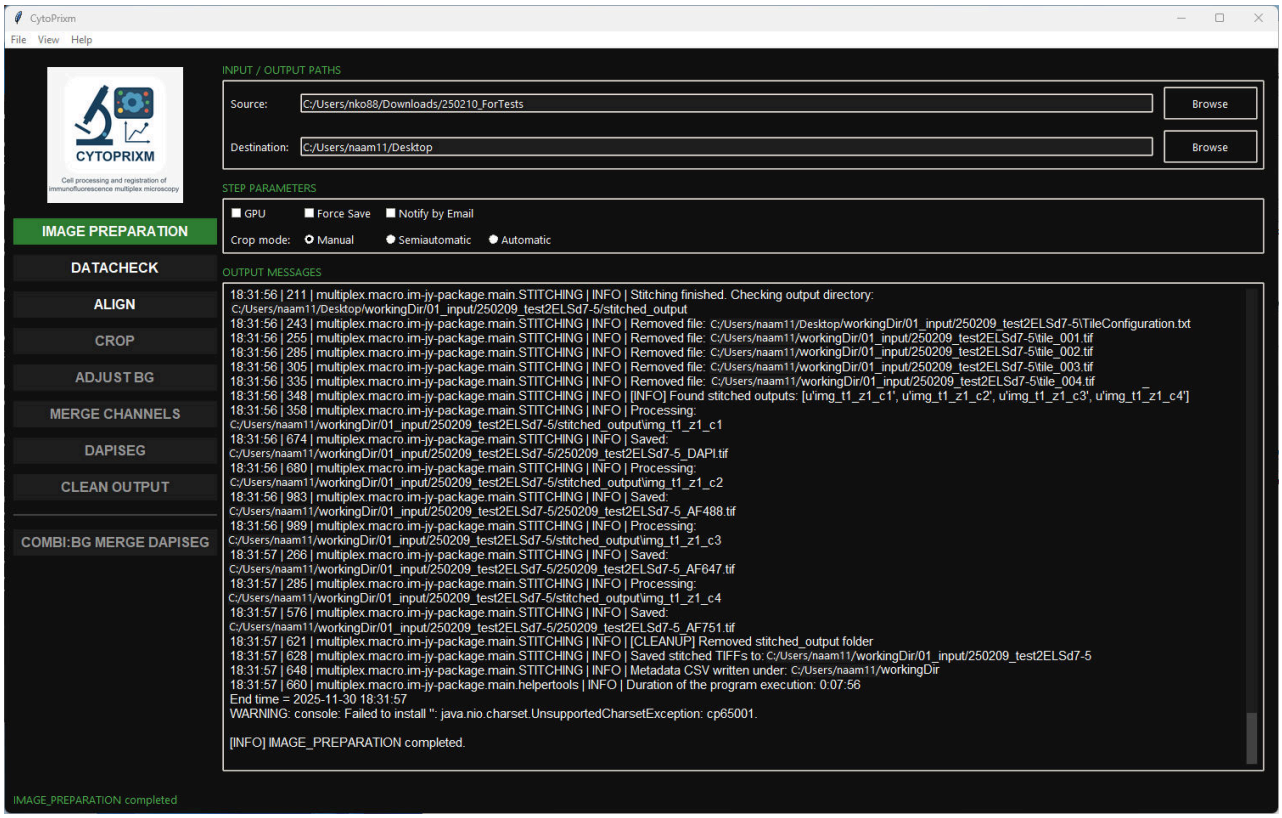

For 2 batches it takes about 1 min and 42s time to process

2.

When you execute the `DATACHECK` step, a window of the graphical user interface (GUI) is loaded showing the input directory path, a table of channels with the used channel cells marked in red for each date and below the output window.

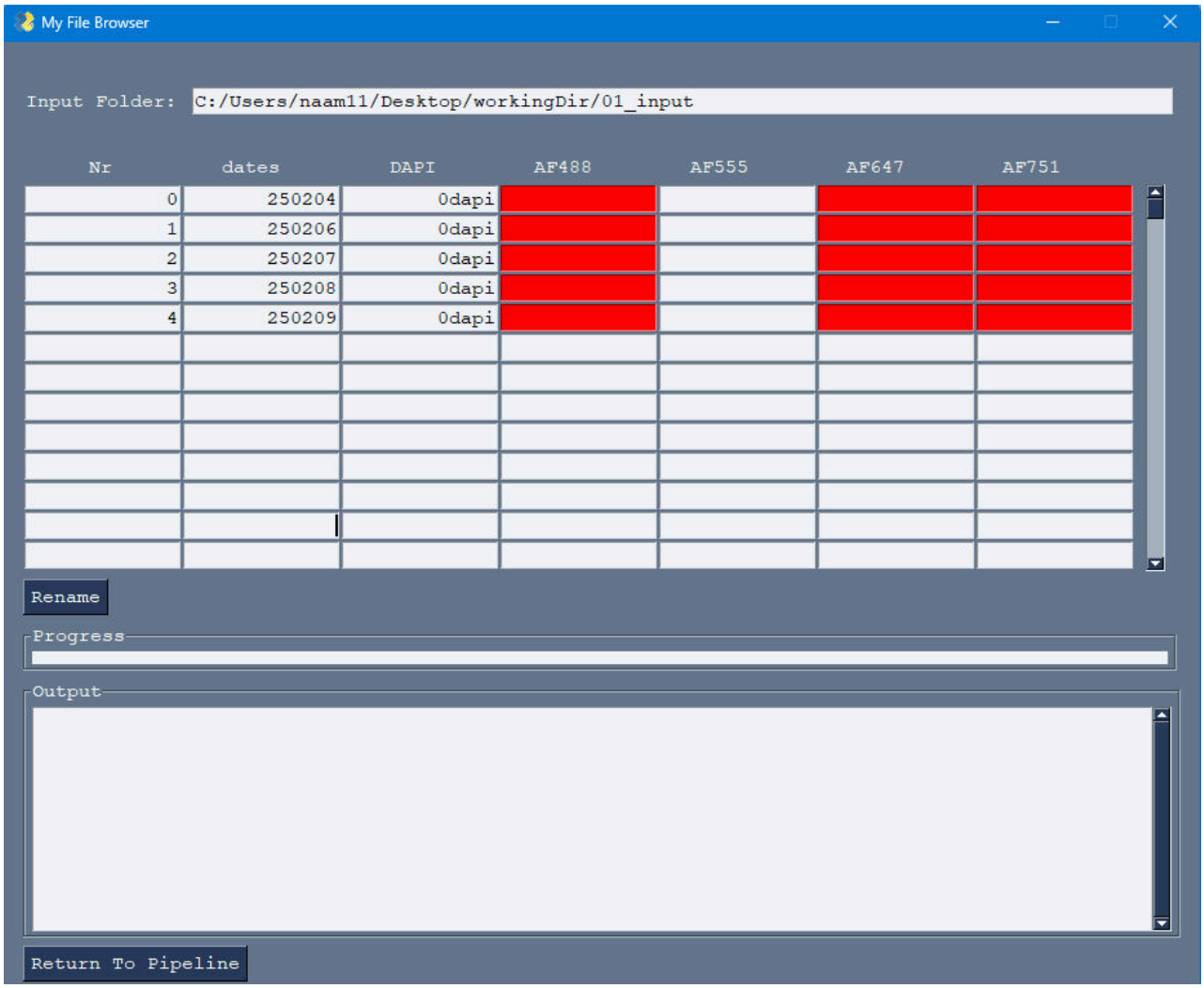

The red cells should be filled by the user with the markers used. Then the user has to click on the RENAME button, which triggers the renaming of the tiff images and their sample ID subfolders. The tiff images will be evaluated, and you will get data in the output window such as how many batches there are for each sample ID, which of them are selected for alignment and which are not (since they have only one batch) and what markers, files and their size each sample ID has. When finished, click on the `Return To Pipeline` button.

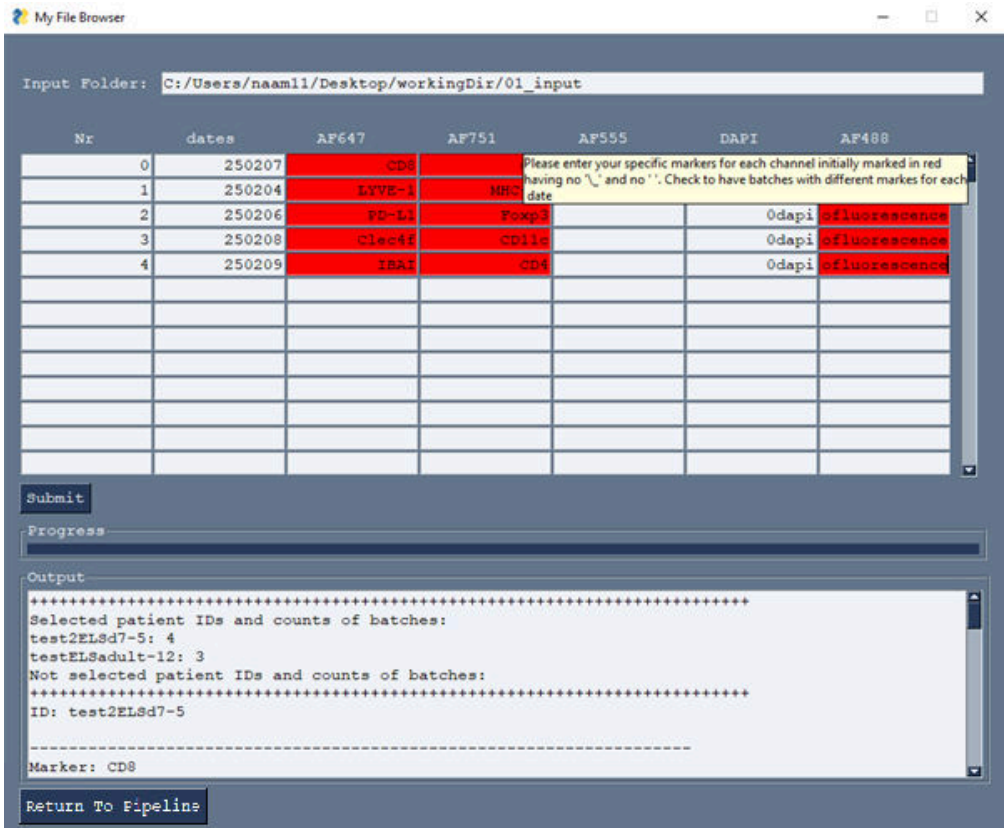

For 2 batches it takes about 3 min time to process (including the time the user edits the table)

3.

The next step is `ALIGN`. In `STEP PARAMETERS` in the `main window` you have to select `ForceSave` option (if you want to overwrite the existing output files), click then on the button `ALIGN`. You will be prompted to set some parameters like `Feature Extraction Model`, `Registration Model` (see https://imagej.net/plugins/register-virtual-stack-slices) and `Background Parameters` (see https://imagej.net/plugins/rolling-ball-background-subtraction) and `autoContrast` (to increase the contrast optimally) for DAPI images. After you have confirmed the selection by clicking `OK` (`Cancel` ends the step), the matching of all directories sorted by `sampleID` is performed. Before alignment the number of tiff images will be adjusted to have the same number of files for each separate sampleID by copies of the DAPI file in the certain folder(s). If the image is corrupted, it will be copied to the error folder `error_subfolder` in the folder `02_alignment`.

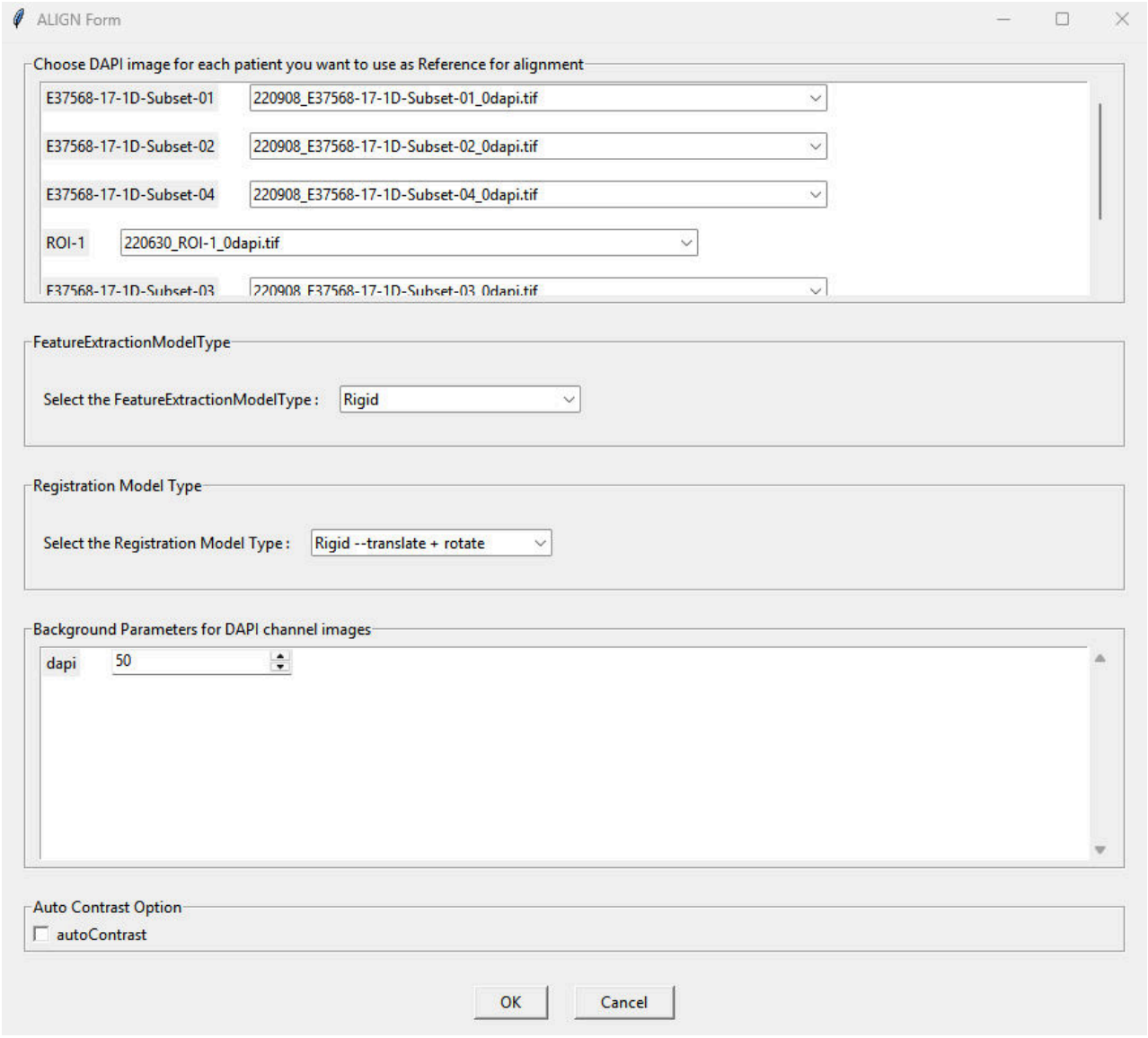

During the alignment three temporary folders `temp`, `out` and `transforms` are created in `02_alignment` if input data are in `01_input_dir`. These temporary subsubfolders are emptied after alignment of each `sampleID` and deleted after the alignment is finished for all `sampleIDs`. After successful alignment the DAPI files are treated with the background subtraction and are combined with other channel images of the certain `sampleID` into a stack and stored in the folder `02_alignment` or the input of misaligned data from `01_input_dir` is copied to the folder `02_01_input_to_precrop`. If the batch (images of one `patientID`) contains image files of one date (`single batch`) only stack of these images will be created without any alignment as it is not needed. Pro batch it takes usually up to 2 hours but may be longer if images have to many features

4.

In the next step `CROP` all image stacks produced during the step `ALIGN` and stored in `02_alignment` are cropped. The user may set `ForceSave` option in the `main window` to overwrite the output data and to select one of the three modes of the cropping step (`Manual Selection`, `Semiautomatic Selection` or `Automatic Selection`). By `Manual Selection` the stack of a certain `sampleID` is loaded, and the user has to set the region of interest manually and then after the confirmation (clicking `Ok` in the `Action required` dialog) it is automatically cropped. By `Semiautomatic Selection` the coordinates of the rectangle frame excluding the black regions of the background will be automatically determined and preset for the user and it can be then adjusted if needed, then after confirmation (clicking `Ok` in the `Action required` dialog) the image files are cropped

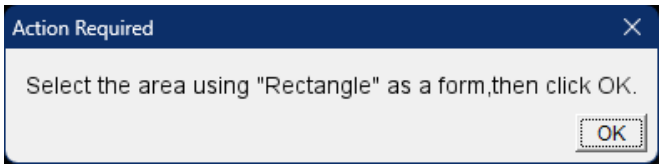

By `Automatic Selection` the coordinates of the rectangle form excluding the black background regions are automatically determined and automatically cut without user intervention. So, after each selection type the stack is cropped and saved in the folder `02_alignment` with the extension `_Cropped`.

The step takes some minutes to process the data

5.

In the next step `ADJUST BG` the background subtraction for the necessary markers takes place (please see https://imagej.net/plugins/rolling-ball-background-subtraction). The user may set `ForceSave` option in the `main window` to overwrite the output data. Thereby the user is prompted to set the background parameter settings. If you press `Ok` (`Cancel` ends the step), the background subtraction applies to the selected images.

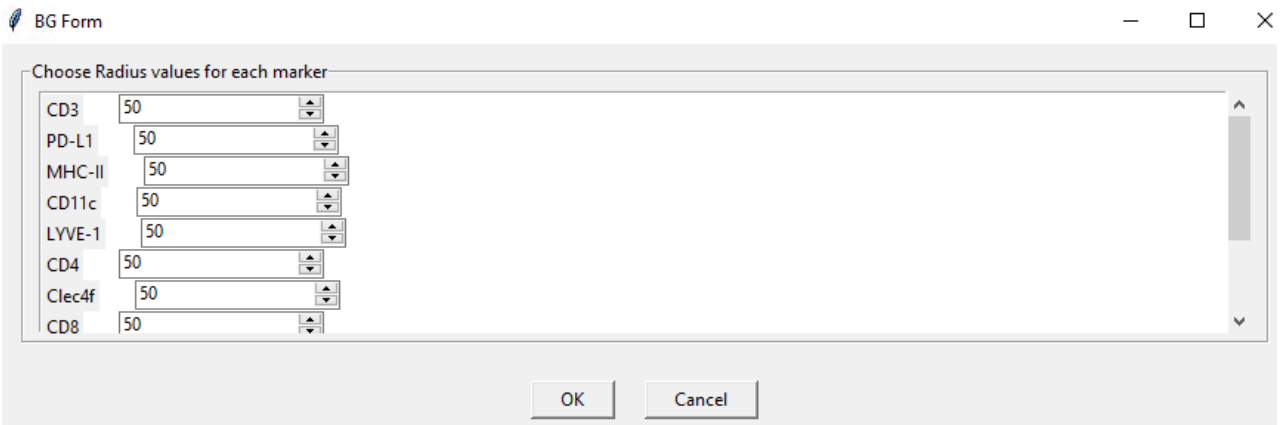

The cropped stacks of images from `02_alignment` are processed and each slice of each aligned stack is saved with (extension: `_background_sub`) and without background subtraction (extension: `_no_background`) in the folder `06_bg_processed`. The step takes some minutes to process the data

6.

For the step `MERGE CHANNELS` the user may set `ForceSave` option in the `main window` to overwrite the output data. In this step, the images from DAPI and other channels from the step background subtraction selected by the user are merged and saved in the folder `07_mergedChannels`. At the beginning, the user is asked to set parameters for the selection of the DAPI image for each sampleID and the images of the channels to be merged with the selected DAPI image. If you press `Ok` (`Cancel` ends the step), the selected marker images are merged with the selected DAPI images.

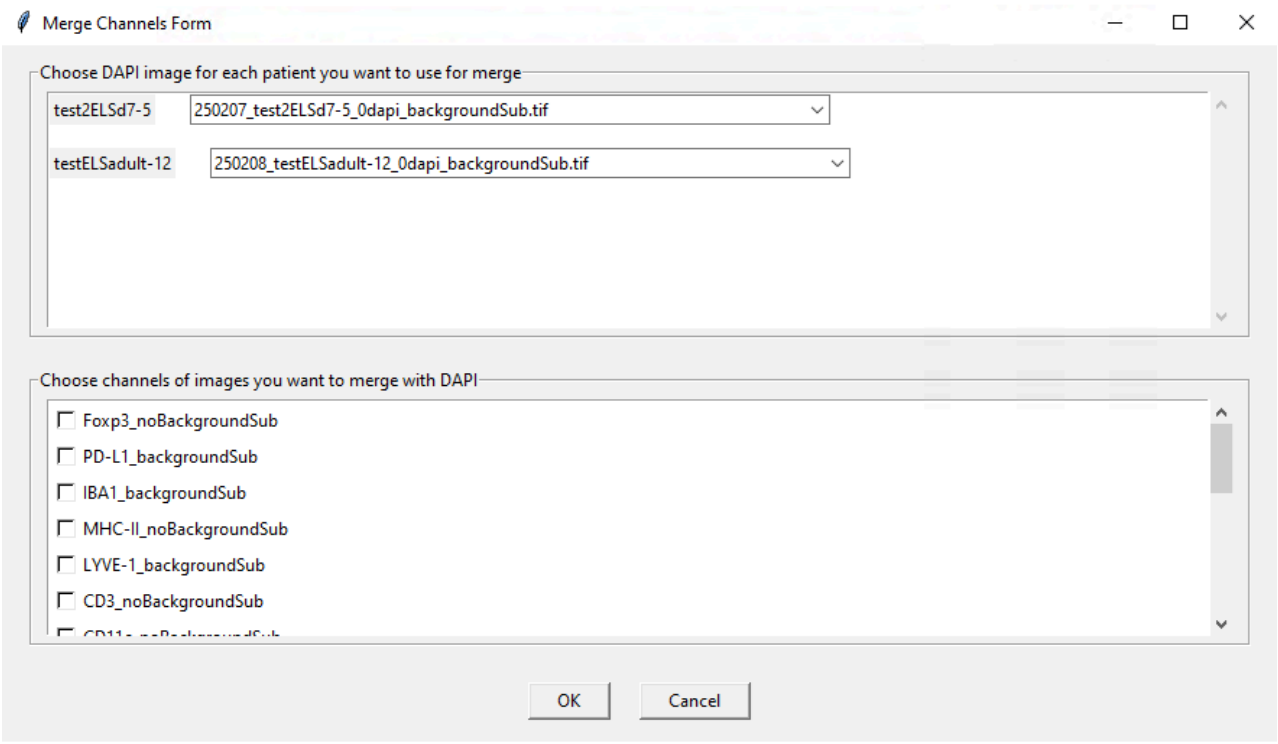

The step takes some minutes to process the data

Releases

No releases published
Create a new release

Packages

No packages published
Publish your first package

Contributors 2

nkon887

adrienguillot

Languages

Jupyter Notebook 82.8% Python 16.7% ImageJ Macro 0.5%

Suggested workflows

Based on your tech stack

Python package Configure

Create and test a Python package on multiple Python versions.

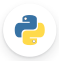

Pylint

Configure

Lint a Python application with pylint.

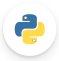

Publish Python Package

Configure

Publish a Python Package to PyPI on release.

More workflows

Dismiss suggestions

7.

The next step is `DAPISEG`. The user may set `ForceSave` option to overwrite the output data and `GPU option` to process the data faster in the `main window`. Thereby the data are put to the correct input form and segmented using the CellSeg package (the scripts and pretrained model from `https://github.com/michaellee1/CellSeg` are adapted to our purpose, contour to entire filling of segmented cells (cell masks), separating neighboured cell masks from each other). Then the segmentation file with the cells of different colour (grayscale gradient) is converted to the binary mask (multiplex), the small holes in the cells are filled and small artifacts removed. Then the masks are resized to have the same size as origin segmented image

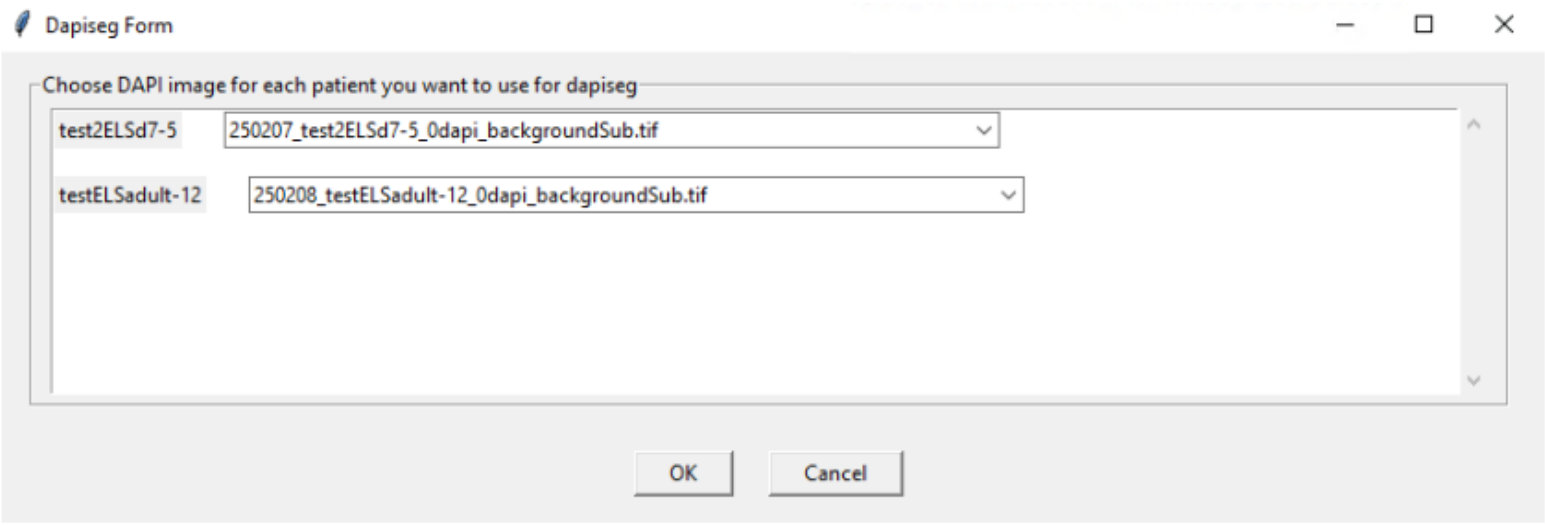

The step takes some minutes to one hour if the image is big

8.

By step `CLEAN OUTPUT` the user is asked (see dialog window below) to confirm to clean redundant intermediate data and store only the results. If the user confirms, all other subfolders in the folder workingDir are deleted and only the final subfolder "06_results_output" and metadata.csv remain in the main folder after this step

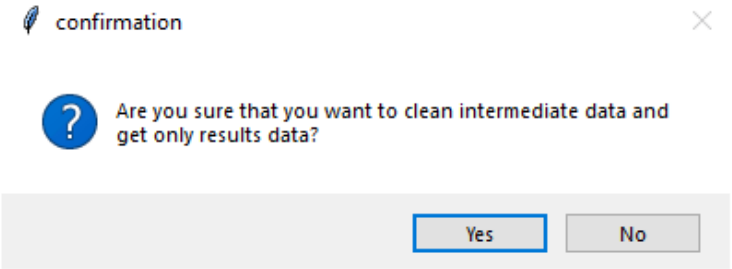

9.

Another possibility is to execute `COMBI:BG MERGE DAPISEG` to do `BG ADJUST`, `MERGE CHANNELS` and `DAPISEG` at once with setting all parameters for these three steps before execution.

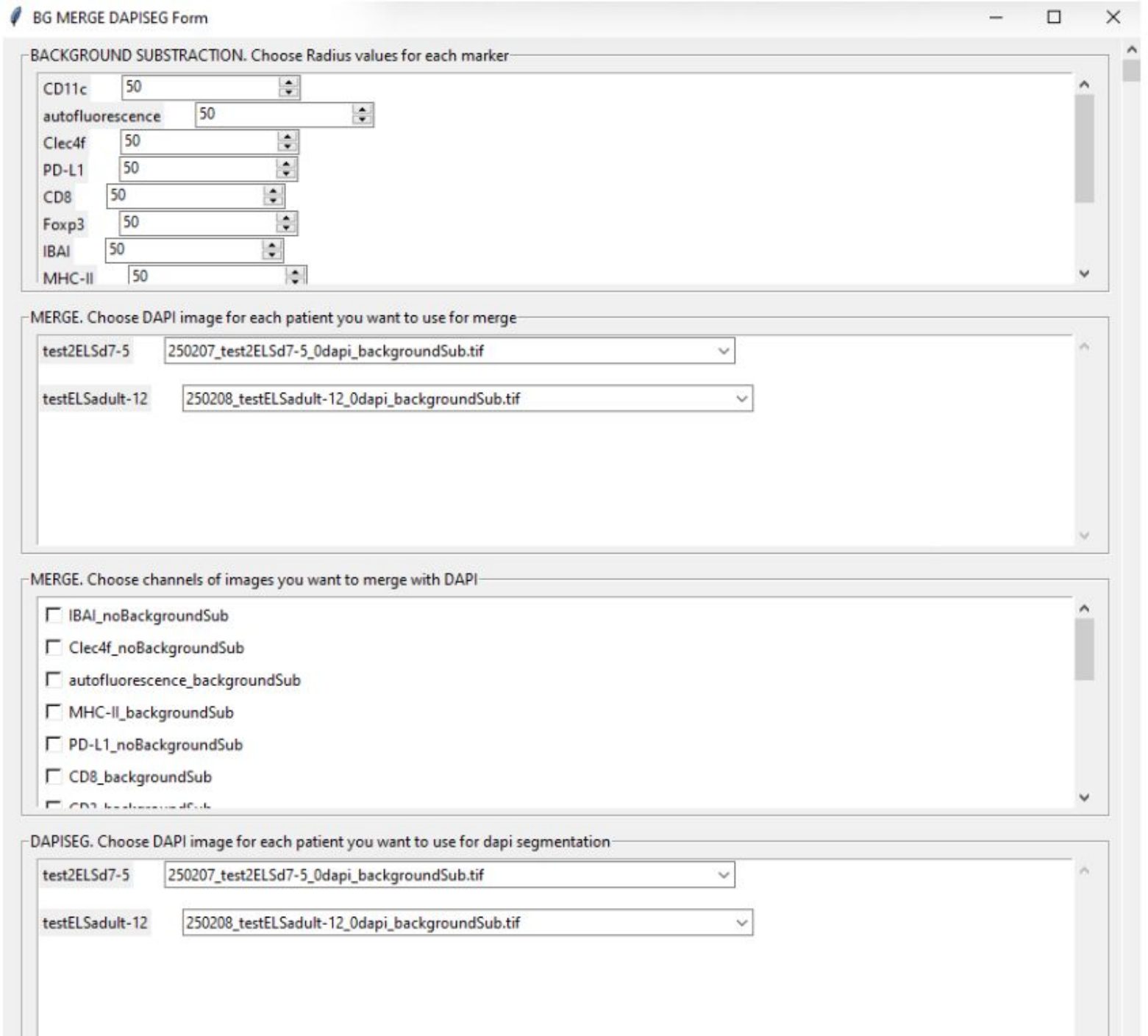

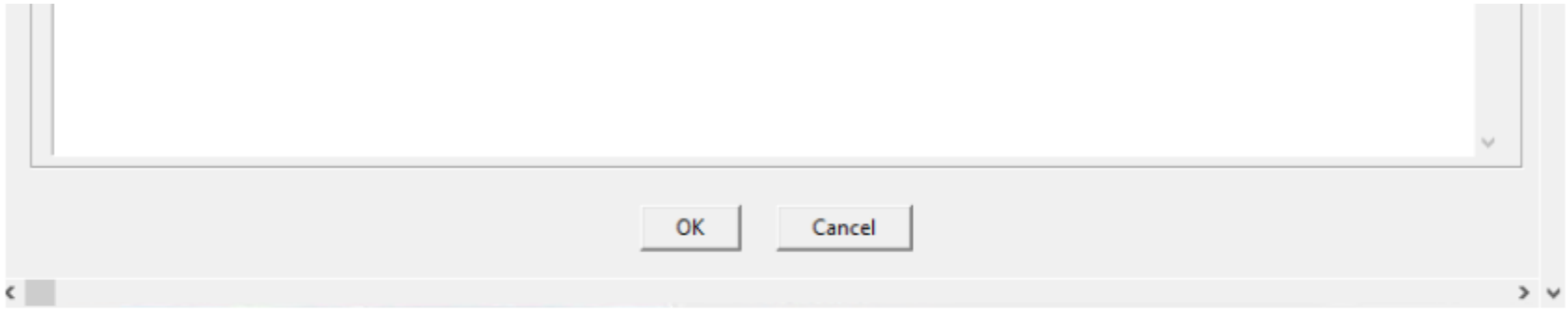

Notes:

- If errors are occurring during the execution, there are outputs on the console of Fiji and in MiniForgePrompt and the history is stored in `logs.log` in the `pipeline/`
- During the execution, there is also an output on the console of Fiji and MiniForgePrompt and is stored in `logs.log`
- The execution times of the steps of image processing are outputted in the end on the console of MiniForgePrompt and in `logs.log`
- Jython/Fiji JARs may trigger Java initialization If the JVM wakes cold (no cache), it takes longer. The pipeline has to scan many files of the envs in the beginning. Large env directories → variable filesystem speed. If the disk cache is cold (e.g., after reboot), it can be slower. If cache is warm, extremely fast
