# Supplementary material for: Unified platform for multiplex immunofluorescence across liver tissues and engineered models
Source: eGastroenterology. 2026 Apr 30;4(2):e100379. doi: 10.1136/egastro-2026-100379 (PMC13141115; doi:10.1136/egastro-2026-100379)
Supplement: Supplementary file 4 [file egastro-2026-100379.reviewer_comments.pdf]

PEER REVIEW HISTORY

eGastroenterology publishes all reviews undertaken for accepted manuscripts. Reviewers are asked to complete a checklist review form and are provided with free text boxes to elaborate on their assessment. These free text comments are reproduced below.

ARTICLE DETAILS

**TITLE (PROVISIONAL)**	A unified platform for multiplex immunofluorescence across liver tissues and engineered models
**AUTHORS**	Franco Leonardi, Bianca<br>Yin, Guo<br>Amiridze, Natalja<br>Lan, Tian<br>Ait Ahmed, Yeni<br>Berger, Hilmar<br>Tacke, Frank<br>Kohlhepp, Marlene<br>Guillot, Adrien

VERSION 1 - REVIEW

**REVIEWER NAME**	***Feng, Dechun***
**REVIEWER AFFILIATION**	National Institutes of Health, National Institue on Alcohol Abuse and Alcoholism
**REVIEWER CONFLICT OF INTEREST**	No competing interest
**AI DISCLOSURE**	No, I have not used AI
**DATE REVIEW RETURNED**	17-Feb-2026

**GENERAL COMMENTS**	In this manuscript, the authors provided a method for multiplex immunofluorescence that can be used for a broad range of samples including FFPE tissue, FFPE organoid, primary cell cultures and liver-on-a-chip models. The workflow is flexible, low-cost and can be adapted by most laboratories with conventional equipment. The manuscript is well organized and provides very useful and detailed information. Here are some comments:<br>1. In Fig.2C, the author mentioned “membrane overlap between two distinct cell types”. Did the author have any solutions to improve the accuracy of cell segmentation when two different types of cells get too close to each other?<br>2. In Fig.4, it will be better to provide an enlarged image for panel D, so the reader can see the detail of the image.<br>3. In Fig.5 and Fig.6, the authors mentioned the autofluorescence of dead cells. It will be better to provide information about which channel to detect autofluorescence. For my understanding, autofluorescence was mainly observed on 488 channels? So which kind of 2nd antibodies do the authors suggest using? 555 and 647?<br>4. In Fig.8, it will be great if the authors can provide more information about the proximity ligation assay. In addition to MHCI-CD8, can it be used for other protein-protein interaction detection?

**REVIEWER NAME**	***Seo, Wonhyo***
**REVIEWER AFFILIATION**	Ewha Womans University, College of Pharmacy

**REVIEWER CONFLICT OF INTEREST**	No competing interest
**AI DISCLOSURE**	No, I have not used AI
**DATE REVIEW RETURNED**	05-Mar-2026

**GENERAL COMMENTS**	The study entitled “A Unified Platform for Multiplex Immunofluorescence Across Liver Tissues and Engineered Models” presents an end-to-end, stripping-based multiplex immunofluorescence (mIF) workflow for liver research which is applicable across FFPE tissue, organoids, primary cell cultures, and a biliary niche-on-a-chip model. It also introduces “CytoPrixm”, an open-source, Python-based tool designed to standardize and accelerate preprocessing of multidimensional mIF images, including shading correction, stitching, background subtraction, and DAPI-based registration. The feasibility of the workflow is demonstrated using mouse and human samples, including integration with a proximity ligation assay (PLA). The authors present this platform as a scalable and easy-to-use approach to high-plex imaging technologies that require specialized and expensive equipment, with the goal of reducing the technical complexity and cost barriers associated with multiplex imaging in the research field of “hepatology”.<br>Overall, the study presents a multiplex immunofluorescence approach highly applicable to diverse experimental contexts, including tissue specimens and primary cell–derived, genetically engineered in vitro systems. The manuscript is clearly well-written and provides useful insights into the application of multiplex immunofluorescence for liver disease research.<br><br>[Comments]<br>1. The concern regarding scalability would be strengthened by providing quantitative evidence demonstrating epitope preservation across staining cycles, reproducibility across experimental batches and operators, and registration accuracy at single-cell resolution. It would be helpful to include quantitative QC results such as normalized intensity retention across cycles, secondary-only imaging after stripping to confirm antibody removal, quantitative measures of image registration accuracy and so on.<br>2. Because sequential (repeated) multiplex imaging can introduce tissue deformation across staining cycles, additional clarification is needed regarding how CytoPrixm addresses this issue.<br>3. For broader adoption, it would be helpful to provide information on the practical throughput and computational requirements of the workflow, including the imaging time per cycle, the total time required for 8–12 staining cycles, the CPU/GPU requirements for running CytoPrixm, and how the runtime scales with tissue area and the number of image tiles.<br>4. Although the study is presented as a unified platform integrating multiplex immunofluorescence with a dedicated analysis workflow, the manuscript primarily emphasizes the immunofluorescence protocol itself. In comparison, the description of CytoPrixm and the overall computational workflow is relatively limited. Please provide additional details regarding the functionality, architecture, and implementation of CytoPrixm.<br>5. The antibody stripping strategy is described; however, please include details on how complete antibody removal between cycles was systematically validated.<br>6. The manuscript presents that several parameters—including sample type (e.g., FFPE vs frozen)—should be considered when

	selecting the analysis method. However, the figure does not provide information on frozen samples. Can CytoPrixm be applied to samples prepared from frozen tissue sections (Were there specific challenges or limitations associated with frozen sections compared to FFPE tissue?)

VERSION 1 – AUTHOR RESPONSE

Comments from the Editorial Office:

1. For this original research, there should be a section to discuss the study limitations.

We apologize that this was missing from our original submission. We now include a "Study limitations" section in the revised version of our manuscript.

2. In the "Liver FFPE sample preparation" of the Materials and Methods section, the manuscript states "Livers from healthy mice," but the mouse strain is not specified. Please indicate the strain used.

We apologize for this and now added the strain of the mice to the Materials and Methods section in our manuscript: "Livers from healthy C57BL/6J mice".

In addition, the source of paraformaldehyde used for fixation ("4% paraformaldehyde, PFA") is not specified. Please provide the manufacturer information for this reagent.

We thank the Editor for spotting this, we now added this information in the Materials and Methods section: After collection, tissues were immediately fixed in 4% paraformaldehyde (PFA, pH 7.4, Morphisto, Frankfurt, Germany).

3. Please ensure that all figures are referred to in the text using the standard format (e.g., "Figure 4B"). If "panel B" is used to describe the content, please also provide the full figure citation (e.g., "Figure 4B") at the end of the sentence in parentheses to meet the formatting requirements for technical review. Specifically, this applies to the citations for Figures 4B, 4C, 4D, 5B, 5C, 6E, 7C, 8B, and 8C.

We now adjusted the references to figure panels according to the Editors requirements.

4. For panels with the same magnification in each figure, at least one representative scale bar should be included, and its length should be clearly indicated.

We thank you for this suggestion and added additional scale bars for the distinct figure panels and magnifications shown through the manuscript figures.

5. The legend of Figure 3 appears to lack a dedicated description for panel C. Please add the legend to describe the content of panel C clearly.

We are sorry for having overlooked this. The Figure 3 legend now includes: "**(C)** Single channel grayscale images from Areas 1 and 2 depicted in (B) are shown.".

6. The figure labels (e.g., "Figure 1", "Figure 2", etc.) should be removed from within the images themselves.

We now removed the figure labels from the single figure files uploaded to the *eGastroenterology* website.

Reviewer(s)' Comments to Author (if any):

Reviewer: 1
Comments to the Author
In this manuscript, the authors provided a method for multiplex immunofluorescence that can be used for a broad range of samples including FFPE tissue, FFPE organoid, primary cell cultures and liver-on-a-chip models. The workflow is flexible, low-cost and can be adapted by most laboratories with conventional equipment. The manuscript is well organized and provides very useful and detailed information.

We want to express our sincere gratitude to the Reviewer’s positive assessment of our manuscript, and their appropriate and greatly appreciated suggestions. We now provide a revised manuscript along with a point-by-point response below.

Here are some comments:

1. In Fig.2C, the author mentioned “membrane overlap between two distinct cell types”. Did the author have any solutions to improve the accuracy of cell segmentation when two different types of cells get too close to each other?
We thank the Reviewer for emphasizing this important aspect. It is indeed challenging to assign segmented objects to one or the other cell type when immunostaining is overlapping for irregular shaped cells as shown in Figure 2C. We now included the following insights from our approach to resolve this issue:

- In the results section: "The lower image represents typical membrane overlaps between those two distinct cell types, which must be considered when performing high-end image analyses. To overcome this issue, in our laboratory we firstly segment nuclei (DAPI) and then assign cell identity based on whether the nuclei are at least 60% overlapping with the respective membrane marker. This is important to prevent falsely concluding on the presence of bi-phenotypic cells; and one should assume it is not relevant to measure LYVE1 staining intensity in $IBA1^{+}$ macrophages. Noteworthy, this approach does not fully resolve issues related to single cell phenotyping, e.g. single cell-resolved lymphocyte expression of polarization profile-specific markers in lymphoid structures with a high cell density and significant membrane contacts, for which specific algorithms must be developed depending on project- and antibody panel-specific considerations.”

- This statement in the new “Study limitations” section: During image analysis, potential artifacts, such as partial membrane overlap between distinct but neighbouring cell populations or autofluorescence, must also be considered in a project- and antibody panel-specific manner to avoid misinterpretation of the results.

2. In Fig.4, it will be better to provide an enlarged image for panel D, so the reader can see the detail of the image.

We are thankful to the Reviewer for this suggestion. We now include images with higher magnification in Figure 4E.

3. In Fig.5 and Fig.6, the authors mentioned the autofluorescence of dead cells. It will be better to provide information about which channel to detect autofluorescence. For my understanding, autofluorescence was mainly observed on 488 channels? So which kind of 2nd antibodies do the authors suggest using? 555 and 647?

We are sorry for the confusion around this aspect of autofluorescence of dead cells. In the revised version of the manuscript, we now indicate all channels for each immunofluorescence image shown in the manuscript (see Figures and Figure legends), which should bring additional insights to other researchers. Furthermore, we added this statement in the result section describing Figure 5: "As mentioned previously, autofluorescence, emitted primarily by dead cells and generally in the 488 and 555 channels, can be detected (…)". The Reviewer rightfully pointed out that autofluorescence is generally observed in the A488 channel, but also in the A555 channel. Hence, in our laboratory we preferably use the Alexa fluor 647 and Alexa fluor 750 fluorophores, which typically give very little to no autofluorescence or background signal when working with liver sections or liver cells.

4. In Fig.8, it will be great if the authors can provide more information about the proximity ligation assay. In addition to MHCI-CD8, can it be used for other protein-protein interaction detection?

We appreciate the interest of the Reviewer for the proximity ligation assay (PLA) technology. The Naveni® in situ proximity ligation technology from Navinci uses an antibody-based approach making it indeed highly versatile. In short, primary antibodies against two target epitopes (i.e., ligand and receptor) are combined with secondary antibodies attached to oligonucleotide arms, that can form together DNA circles if they are in close proximity, meaning that the epitopes recognized by the primary antibodies are close enough to assume ligand-receptor interactions. These DNA circles are then amplified, and fluorescent probes are added to create a highly sensitive and specific signal. As the technology relies on primary and secondary antibodies, it can be applied to any protein-protein interaction target for which good primary antibodies are available. The manufacturer also offers kits for detecting PD1/PD-L1 interactions as well as flexible kits for any target of interest to be tailored to the investigators' needs (https://navinci.se/). This has been further discussed in the revised manuscript.

Reviewer: 2

Comments to the Author

The study entitled "A Unified Platform for Multiplex Immunofluorescence Across Liver Tissues and Engineered Models" presents an end-to-end, stripping-based multiplex immunofluorescence (mIF) workflow for liver research which is applicable across FFPE tissue, organoids, primary cell cultures, and a biliary niche-on-a-chip model. It also introduces "CytoPrixm", an open-source, Python-based tool designed to standardize and accelerate preprocessing of multidimensional mIF images, including shading correction, stitching, background subtraction, and DAPI-based registration. The feasibility of the workflow is demonstrated using mouse and human samples, including integration with a proximity ligation assay (PLA). The authors present this platform as a scalable and easy-to-use approach to high-plex imaging technologies that require specialized and expensive equipment, with the goal of reducing the technical complexity and cost barriers associated with multiplex imaging in the research field of "hepatology".

Overall, the study presents a multiplex immunofluorescence approach highly applicable to diverse experimental contexts, including tissue specimens and primary cell–derived, genetically engineered in vitro systems. The manuscript is clearly well-written and provides useful insights into the application of multiplex immunofluorescence for liver disease research.

We thank the Reviewer for their evaluation of our work and for their constructive comments. We hope to have addressed all the points below.

[Comments]

1. The concern regarding scalability would be strengthened by providing quantitative evidence demonstrating epitope preservation across staining cycles, reproducibility across experimental batches and operators, and registration accuracy at single-cell resolution. It would be helpful to include quantitative QC results such as normalized intensity retention across cycles, secondary-only imaging after stripping to confirm antibody removal, quantitative measures of image registration accuracy and so on.

We sincerely thank the Reviewer for this valuable suggestion. We agree that quantitative assessment of epitope preservation, antibody stripping efficiency, and image registration accuracy would further strengthen the evaluation of the antibody stripping-based multiplex immunofluorescence method for large-scale quantitative analyses. Due to the limited revision timeframe and the primary scope of the manuscript, we were not able to perform a comprehensive quantitative analysis across multiple cycles, operators, and batches. However, we want to outline several steps we implemented to ensure robustness of the approach. In our workflow, fluorescence intensities are analyzed in a relative and normalized manner within large scans or across samples processed within the same experimental batch, which reduces the impact of inter-sample and inter-operator variability.

Regarding epitope preservation, the 2-Mercaptoethanol/SDS-based stripping method used here has previously been shown to preserve antigenicity and can even enhance signal for certain epitopes, likely due to its antigen retrieval-like effect (PMID: 24794148). Consistent with this, we observed stable or increased signal intensity for several markers across staining cycles in our experiments.

To support reproducibility and registration accuracy, we refer to our previous work (PMID: 32872334), where tissue integrity and single-cell level image registration were validated on FFPE tissues using a similar workflow. Based on our experience from the several publications that followed and in which we applied multiplex immunofluorescence to diverse FFPE tissues, the main source of variability arises from tissue quality (e.g., fixation duration, storage conditions, and section handling) rather than staining or stripping variability, and this indeed generates barriers to upscaling the protocol to very large sample cohorts. To further highlight those considerations, we now include practical recommendations regarding section preparation and storage (i.e., use of freshly cut sections or storage at 4°C) in our revised manuscript.

Yet, we fully agree with the Reviewer that a systematic quantitative QC framework and normalisation of immunofluorescence images (e.g., intensity retention, exposure time variations, stripping efficiency controls, and registration metrics) would be highly valuable to the field, especially in the era of multiplex imaging. We plan to include such analyses in a future study,

possibly one that would solely explore those aspects considering the extensive and many layers of analyses required to robustly address such a vast question.

2. Because sequential (repeated) multiplex imaging can introduce tissue deformation across staining cycles, additional clarification is needed regarding how CytoPrixm addresses this issue.
Thank you for asking this question. The default algorithm used for image registration in CytoPrixm is the “rigid alignment”, yet the user has the possibility to select the “affine” algorithm which would consider possible tissue deformation. Using the default “rigid” approach, the software does not alter the input images beyond translation and rotation to match the sequential days of imaging. For all images shown in this manuscript, we only used the “rigid” alignment algorithm. In our hands, this algorithm is sufficient to obtain aligned images as shown in the different figures displaying the DAPI overlays and thus, demonstrates that tissue morphology is not deformed through the successive staining/stripping cycles. In cases where there is tissue damage or detachment due to poor tissue preservation or technical errors during the procedure, a manual pre-cropping of the input images can be performed to remove the damaged tissue areas prior to image alignment in CytoPrixm. In an earlier version of the CytoPrixm software, this step of pre-cropping was implemented (data not shown) as an additional stage but we later decided to deactivate it, as it was not routinely needed and was making the interface and user experience more complex. In the unlikely event tissues would be deformed, we now encourage the users to either use the “affine” function, pre-crop manually their images prior to loading them into CytoPrixm, or to crop the problematic areas in the “cropping” step after alignment, as described in the user’s manual now provided as Supplementary material.

3. For broader adoption, it would be helpful to provide information on the practical throughput and computational requirements of the workflow, including the imaging time per cycle, the total time required for 8–12 staining cycles, the CPU/GPU requirements for running CytoPrixm, and how the runtime scales with tissue area and the number of image tiles.

We greatly appreciate the suggestion from the Reviewer, this is indeed very relevant information that will guide future users to set-up their working environment. In the revised manuscript, we now attach a step-by-step guide on how to install and run the CytoPrixm software. In addition, we now provide in the Supplementary Materials some additional information on two systems on which we ran comparative rounds of image processing using CytoPrixm. Details about the imaging datasets and processing times are shown in Supplementary Materials and below for the Reviewer’s convenience.

Supplementary Table 4: Imaging metadata for the sample dataset used in this comparison. This imaging dataset corresponds to a 155-tile scan which was acquired using a Plan-Apochromat 20x/0.8 M27 objective.

Cycle #	Exposure time #1 DAPI (ms)	Exposure time #2 AF488 (ms)	Exposure time #3 AF647 (ms)	Exposure time #4 AF750 (ms)
1	2.5	24	150	250

2	3.0	24	180	300
3			100	
4			42	
5			60	
6			60	
7			60	
8			30	

Supplementary Table 5: Hardware requirements and recommendations for running CytoPrixm on large-scale images.

**CPU requirement for CytoPrixm**	Multi-core CPU recommended; minimum 8 cores, practical 16+ cores
**RAM requirement**	Minimum 32 GB, practical 64–128 GB for large datasets
**GPU requirement**	Optional for general preprocessing; recommended for DAPI segmentation
**Recommended GPU**	NVIDIA GPU with at least 8–12 GB VRAM; practical 16+ GB for large batches
**Disk storage requirement**	High; typically several hundred GB to multiple TB per project
**Disk speed**	SSD strongly recommended
**Runtime scaling with tissue area**	linear
**Runtime scaling with number of image tiles**	linear

**Runtime scaling with number of staining cycles**	linear
**Alignment cost scaling**	Increases with image size and number of cycles
**Segmentation cost scaling**	Strong increase with total pixel count

Supplementary Table 6: Comparison of CytoPrixm processing times using two computers, on a large imaging dataset. We used two computers: The PC was configured with an Intel i7-8565U CPU (4 cores, 8 threads) and 16 GB of RAM; no GPU was specified. The laboratory workstation was equipped with an Intel Xeon Gold 6244 CPU (8 cores, 16 threads, ~3.6 GHz), 192 GB of RAM, a 27.29 TB RAID array and a 477 GB SSD for storage, and an NVIDIA Quadro RTX 5000 GPU with 16 GB of memory.

	**PC**	**Lab workstation**
**Image preparation**	5 h 51 min	3 h 05 min 59 s
**Datacheck**	1 min 55 s	2 min 3s
**Alignment**	47 min 47 s	1 h 48 min 35 s
**Crop (automatic)**	3 min 54 s	7 min 26 s
**Adjust background**	56 min 34 s	44 min 05 s
**Merged channels**	24 min 59 s	41 min 01 s
**DAPI segmentation**	13 hours	1 h 15 min 8 s
**Clean output**	12 min 54 s	23 min 17 s

4. Although the study is presented as a unified platform integrating multiplex immunofluorescence with a dedicated analysis workflow, the manuscript primarily emphasizes the immunofluorescence protocol itself. In comparison, the description of CytoPrixm and the overall computational workflow is relatively limited. Please provide additional details regarding the functionality, architecture, and implementation of CytoPrixm.

In our original submission, we provided a link to an external resource on which the user could download a detailed, step-by-step guide on how to download, install and use the CytoPrixm software. We are sorry if this was not clearly stated. In the revised manuscript, we now provide the software and supporting files into the Zenodo platform

(https://zenodo.org/records/19059527). In addition, we now provide as Supplementary Material to this manuscript the step-by-step CytoPrixm guide. In addition, we revised Figure 1B to hopefully clarify the software architecture. We hope this describes in sufficient details all aspects related to the functionalities, structure and implementation of CytoPrixm.

5. The antibody stripping strategy is described; however, please include details on how complete antibody removal between cycles was systematically validated.

We agree that the complete removal of antibodies is essential for obtaining good and reliable results when using this method of cyclic multiplex immunofluorescence. Based on our experience, how well the antibody stripping performs generally depends on the abundance of the target protein and on the performance of a specific antibody product. Therefore, primary antibodies are initially tested empirically to evaluate how well they can be stripped and this is taken into consideration for the antibody panel design (i.e., antibody staining sequence and antibody dilutions). Noteworthy, antibody stripping may be repeated several times for particularly challenging situations, but this remains exceptional in our experience. One practical solution is to design the antibody staining sequence in a way that challenging-to-strip antibodies will be used toward the end of the staining sequence, and that epitopes that are clearly distinguishable from one another are used in subsequent cycles to identify remaining signal from one cycle to another. As shown in the present manuscript, complete antibody removal is validated from one day to another through the absence of the previous signal on the images generated the following days.

6. The manuscript presents that several parameters—including sample type (e.g., FFPE vs frozen)—should be considered when selecting the analysis method. However, the figure does not provide information on frozen samples. Can CytoPrixm be applied to samples prepared from frozen tissue sections (Were there specific challenges or limitations associated with frozen sections compared to FFPE tissue?)

7. We thank the Reviewer for raising this important point. Unfortunately, the sequential multiplex staining protocol cannot be applied to frozen tissue as this material cannot withstand the chemical antibody stripping procedure. Hence, the CytoPrixm software cannot be provided with images derived from multiplex immunofluorescence performed on frozen tissues. This point has been further clarified in the revised discussion of the manuscript.

VERSION 2 – REVIEW

**REVIEWER NAME**	***Feng, Dechun***
**REVIEWER AFFILIATION**	National Institutes of Health, National Institue on Alcohol Abuse and Alcoholism
**REVIEWER CONFLICT OF INTEREST**	No competing interest
**AI DISCLOSURE**	No, I have not used AI
**DATE REVIEW RETURNED**	24-Mar-2026

**GENERAL COMMENTS**	I am satisfied with the revised version

**REVIEWER NAME**	***Seo, Wonhyo***
**REVIEWER AFFILIATION**	Ewha Womans University, College of Pharmacy

**REVIEWER CONFLICT OF INTEREST**	No competing interest
**AI DISCLOSURE**	No, I have not used AI
**DATE REVIEW RETURNED**	27-Mar-2026

**GENERAL COMMENTS**	All concerns have been well addressed.
